# Broccoli for the brain: a review of the neuroprotective mechanisms of sulforaphane

**DOI:** 10.3389/fncel.2025.1601366

**Published:** 2025-07-04

**Authors:** Riley N. Bessetti, Karen A. Litwa

**Affiliations:** ^1^Department of Anatomy and Cell Biology, East Carolina University, Greenville, NC, United States; ^2^East Carolina Diabetes and Obesity Institute, East Carolina University, Greenville, NC, United States

**Keywords:** sulforaphane, Nrf2, autism spectrum disorder (ASD), epilepsy, neuroprotection

## Abstract

Sulforaphane, a phytochemical abundant in the sprouts of cruciferous vegetables, protects plants during a critical period of development. Through sulforaphane’s ability to activate the mammalian Nuclear Factor Erythroid 2-related Factor 2 (NRF2) pathway, these beneficial properties extend beyond plants. Our current review explores emerging neuroprotective mechanisms of sulforaphane and their relation to neurological disorders. Primarily, we discuss the ability of sulforaphane to mitigate oxidative stress and prevent neuroinflammation. Given sulforaphane’s ability to activate multiple cytoprotective mechanisms, sulforaphane is emerging as a promising therapeutic for multiple neurodegenerative and neurodevelopmental disorders. In this review, we highlight current clinical trials in neurological disorders and conclude by discussing therapeutic opportunities and challenges for sulforaphane. Together, preclinical models and clinical trials highlight emerging themes of sulforaphane-mediated neuroprotection, including hormetic responses that depend upon the cell/tissue, neurological condition, insult, and developmental stage. In particular, low sulforaphane doses consistently exhibit beneficial effects in preclinical neuronal cell cultures models and avoid cytotoxic effects of higher sulforaphane doses. These factors will be important considerations in informing therapeutic use of sulforaphane.

## 1 Introduction to beneficial properties of sulforaphane from plants to animals

Phytochemicals are bioactive compounds naturally produced by plants as mechanisms evolved to protect the plant from environmental stressors, pathogens, and predators. As such, these compounds are non-essential to humans, but in many instances, have demonstrated beneficial effects for human health. Phytochemicals provide protection during sensitive developmental periods, allowing the plant to reach maturation and continue to propagate (Pawase et al., 2024). For example, saponin is a natural surfactant found in leaves, roots, and seeds of many plants, such as quinoa, legumes, and tomatoes. Saponin’s powerful anti-pathogenic properties protect seeds from fungus and insects (Sparg et al., 2004; Zaynab et al., 2021). Other phytochemicals, such as sulforaphane, are upregulated in response to environmental stressors. Sulforaphane is produced upon plant damage and activates production of extracellular reactive oxygen species (ROS) to neutralize external threats, such as pathogens (Rahman et al., 2022; Arruebarrena Di Palma et al., 2022; Qu et al., 2017). Notably, sulforaphane-induced ROS production is specific to plants; sulforaphane promotes extracellular ROS production through activation of the plant-specific Nicotinamide Adenine Dinucleotide Phosphate (NADPH) oxidase, respiratory burst oxidase homologue D (RBOHD) as part of the plant’s innate immune response (Arruebarrena Di Palma et al., 2022; Lee et al., 2020). While increased ROS production can also be detrimental to the plant, at subtoxic doses, sulforaphane also activates the plant’s host defense mechanisms by inducing expression of stress response genes, such as heat shock proteins (Ferber et al., 2020). Heat shock proteins promote expression of antioxidant genes to neutralize oxidative stress within the plant (Ul Haq et al., 2019). This allows sulforaphane to promote plant survival while also combating external threats. Perhaps, not surprisingly, sulforaphane is most abundant in the sprouts of cruciferous vegetables, where it facilitates survival and development into mature plants (Fahey et al., 1997).

In mammals, sulforaphane can similarly upregulate stress-induced signaling pathways, including the Nuclear Factor Erythroid 2-related Factor 2 (NRF2) transcriptional pathway, which is conserved across mammalian species, but absent from plants (Gacesa et al., 2016; Fuse and Kobayashi, 2017). Sulforaphane activates NRF2 transcriptional machinery to upregulate the expression of antioxidant genes and phase II detoxifying enzymes. As sulforaphane will be the focus of our review, we will first present detailed information on sulforaphane and the NRF2 pathway before exploring its beneficial effects in neurological disorders.

## 2 Discovery of sulforaphane and the NRF2-ARE pathway

**Figure 1:**
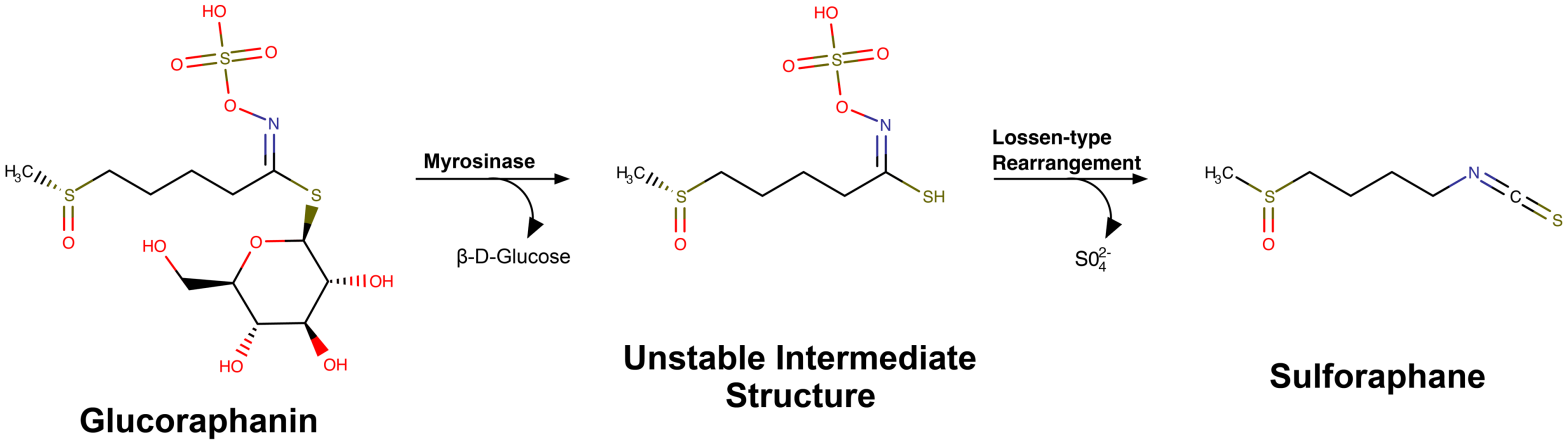
Chemical structure and conversion of glucoraphanin into sulforaphane. MarvinSketch was used to draw substructures and reactions, Marvin Sketch 24.3.2, Chemaxon (https://chemaxon.com) under individual research license by Riley Bessetti.

Sulforaphane is an isothiocyanate, derived from the precursor glucoraphanin (Fahey et al., 1997; Yagishita et al., 2019). In response to plant damage, release of the myrosinase enzyme converts glucoraphanin into sulforaphane (Matusheski et al., 2004; Yagishita et al., 2019; Cai et al., 2020) (Figure 1). The precursor, glucoraphanin, is found in high abundance in cruciferous vegetables and was initially discovered due to researchers’ interest in the chemistry of sulfur-containing natural products. The significance of these plant-derived chemicals in human health continued to grow when these compounds were identified as having potential chemotherapeutic effects. Fascinatingly, the cultivation of these glucoraphanin-rich crops for medicinal purposes can be observed throughout history and even traced back to recordings from ancient civilizations (Fenwick et al., 1983). As we will discuss below, glucoraphanin-rich preparations with active myrosinase enzyme are often used today in clinical trials as glucoraphanin precursor is more stable than sulforaphane (Yagishita et al., 2019) (Table 1).

**Table 1:** ASD clinical trials.

Study design ClinicalTrials.gov ID	Age groups, gender, enrolment	SFN or precursor	Dosage	Measured outcomes/findings	Citation
Randomized, phase 2, double-blind, 18-week study NCT01474993	13–30 years, Male, *N* = 44	SFN-rich encapsulated Broccoli Sprout Extract (each gelcap containing ~ 250 mg SFN-rich Broccoli Sprout Extract, equivalent to ~ 50 μmol of SFN)	Dose dependent on body weight: 50 μmol (one capsule) of sulforaphane for <100 lb., 100 μmol (two capsules) for 101–199 lb., and 150 μmol (three capsules) for >200 lb	Aberrant Behavior Checklist sulforaphane vs. placebo total score improvement: 4 weeks *p =* 0.035; 10 weeks *p =* 0.002; 18 weeks *p* < 0.001 Irritability: sulforaphane vs. placebo improvement significant at week 10 and 18 Lethargy: sulforaphane vs. placebo improvement significant at week 10 and 18 Social Responsiveness Scale sulforaphane vs. placebo improvement: 18 weeks *p =* 0.017 Clinical Global Impression-Improvement sulforaphane 18 weeks vs. placebo 18 weeks: Social interaction *p =* 0.007; Aberrant/abnormal behavior *p =* 0.014; verbal communication *p =* 0.015	Singh et al. (2014)
Phase 3, open-label, 12-week study NCT02654743	7–21 years, 12 male 3 female, *N* = 15	Glucoraphanin dietary supplement (Avmacol®)	~ 2.5 μmol glucoraphanin per lb	Aberrant Behavior Checklist Improvement from baseline total score: 1 month *p =* 0.02 Social withdrawal improvement from baseline: 1 month *p =* 0.001; 3 month *p =* 0.02 Stereotypy improvement from baseline: 1 month *p =* 0.02 Social Responsiveness Scale Improvement from baseline total score: month 3 *p =* 0.03 Communication improvement from baseline: month 3 *p =* 0.005 Motivation improvements from baseline: month 1 *p =* 0.003 and month 3 *p =* 0.001 Correlation of urinary metabolites with ABC and SRS scores	Bent et al. (2018)
Randomized, double-blind, 36-week study	3–7 years, 24 male 4 female, *N* = 28	SFN-rich extract BroccoPhane® broccoli sprout powder and red radish sprout powder	50 μmol SFN per day	Sulforaphane demonstrated no clinical improvement in the following evaluations: Aberrant Behavior Checklist Social Responsiveness Score Autism Diagnostic Observation Schedule-2 Scores Parent’s Impression Scale of ASD symptoms	Magner et al. (2023)
Randomized, double-blind, 12-week study NCT02879110	3–15 years, *N* = 108	Glucoraphanin dietary supplement (Avmacol®)	Two tablets/day for 10–29 lb., three tablets/day for 30–49 lb., four tablets/day for 50–69 lb., six tablets/day for 70–89 lb., seven tablets/day for 90–109 lb., and eight tablets/day for 110–130 lb	Autism Behavior Checklist No significant improvement Social Responsiveness Scale No significant improvement Repetitive Behavior Scale No significant improvement Clinical Global Impression Scale No significant change in severity Estimated improvement score sulforaphane vs. placebo: 8 weeks and 12 weeks *p* < 0.001 OSU Autism Rating Scale-DSM-IV (for all patients) Total average score improved: 8 weeks and 12 weeks *p* < 0.01 and overall analysis *p =* 0.002 Impaired social interaction improved: 8 weeks *p* < 0.01 and 12 weeks *p* < 0.001 and overall analysis *p* < 0.001 Communication barriers improved: 8 weeks *p* < 0.05 and 12 weeks *p* < 0.01 and overall analysis *p =* 0.002	Ou et al. (2024)
Randomized, double-blind, 10-week study	4–12 years, 40 male 20 female, *N* = 60	Risperidone plus sulforaphane	Daily 50 μmol (≤45 kg) or 100 μmol (>45 kg)	Autism Behavior Checklist-Community Overall score not significantly changed Irritability significantly changed from baseline at week 10: Risperidone plus sulforaphane vs. Risperidone plus placebo *p =* 0.001 Hyperactivity/Noncompliance significantly changed from baseline at week 10: Risperidone plus sulforaphane vs. Risperidone plus placebo *p =* 0.015	Momtazmanesh et al. (2020)
Randomized, double-blind, 15-week study followed by open-label, 15-week study and 6-week no-treatment extension (washout) NCT02561481	3–12 years, *N* = 45	Glucoraphanin dietary supplement (Avmacol®)	Daily 30–50 lbs., 3 tablets (45 μmol/day); 50–70 lbs., 4 tablets (60 μmol/day); 70–90 lbs., 6 tablets (90 μmol/day); 90–110 lbs., 7 tablets (105 μmol/day); 110–130 lb., 8 tablets (120 μmol/day)	Aberrant Behavior Checklist Significant changes in total score from baseline and placebo after 15 weeks of exposure Social Responsiveness Scale Significant changes in total score only from baseline after 15 weeks of exposure Ohio Autism Clinical Global Impressions Scale No significant improvement of total score Week 22 social interaction severity sulforaphane vs. placebo *p =* 0.007 Reduced expression of IL-6 and TNFα mRNA at 15 weeks sulforaphane vs. placebo *p* < 0.05	Zimmerman et al. (2021)

While glucoraphanin-rich crops have been cultivated for centuries, cancer research was a driving force in the discovery of sulforaphane. In the 1970s and 80s, epidemiologic evidence uncovered a correlation between increased consumption of vegetables within the *Cruciferae* and *Brassica* families and reductions to an individual’s colon cancer risk (Graham et al., 1978; Colditz et al., 1985). This correlation led to the discovery of phase II detoxification enzymes as a mechanism of resilience against carcinogens (Prochaska et al., 1985). Ultimately in the early 1990’s, Dr. Paul Talalay and researchers at Johns Hopkins University linked these findings (Prochaska et al., 1992) and identified sulforaphane, isolated from broccoli, as a potent inducer of phase II detoxification enzymes including glutathione-S-transferase (GST) and NAD(P)H oxidoreductase 1 (NQO1) (Zhang et al., 1992). During this era of research, other groups focused on the molecular machinery responsible for phase II detoxification enzyme gene expression. This led to the discovery of the short gene regulatory regions called antioxidant response elements (AREs) that are required for xenobiotic and oxidative stress-induced expression of phase II detoxification enzymes (Rushmore and Pickett, 1990; Rushmore et al., 1991; Xie et al., 1995). Subsequently, Nuclear Factor Erythroid 2-related Factor 2 (NRF2) was identified as a transcription factor involved in regulating gene expression at these ARE sequences (Itoh et al., 1997; Ma et al., 2004). Together, these foundational studies elucidated NRF2-ARE-induced genes involved in glutathione production and activity; the detoxification of reactive oxygen species, nitrogen species (ROS/RNS) and xenobiotics; and NADPH production. While these studies elucidated key mechanisms of cellular antioxidant and cytoprotective transcriptional activation, the mechanisms controlling NRF2 activity were still a mystery.

**Figure 2:**
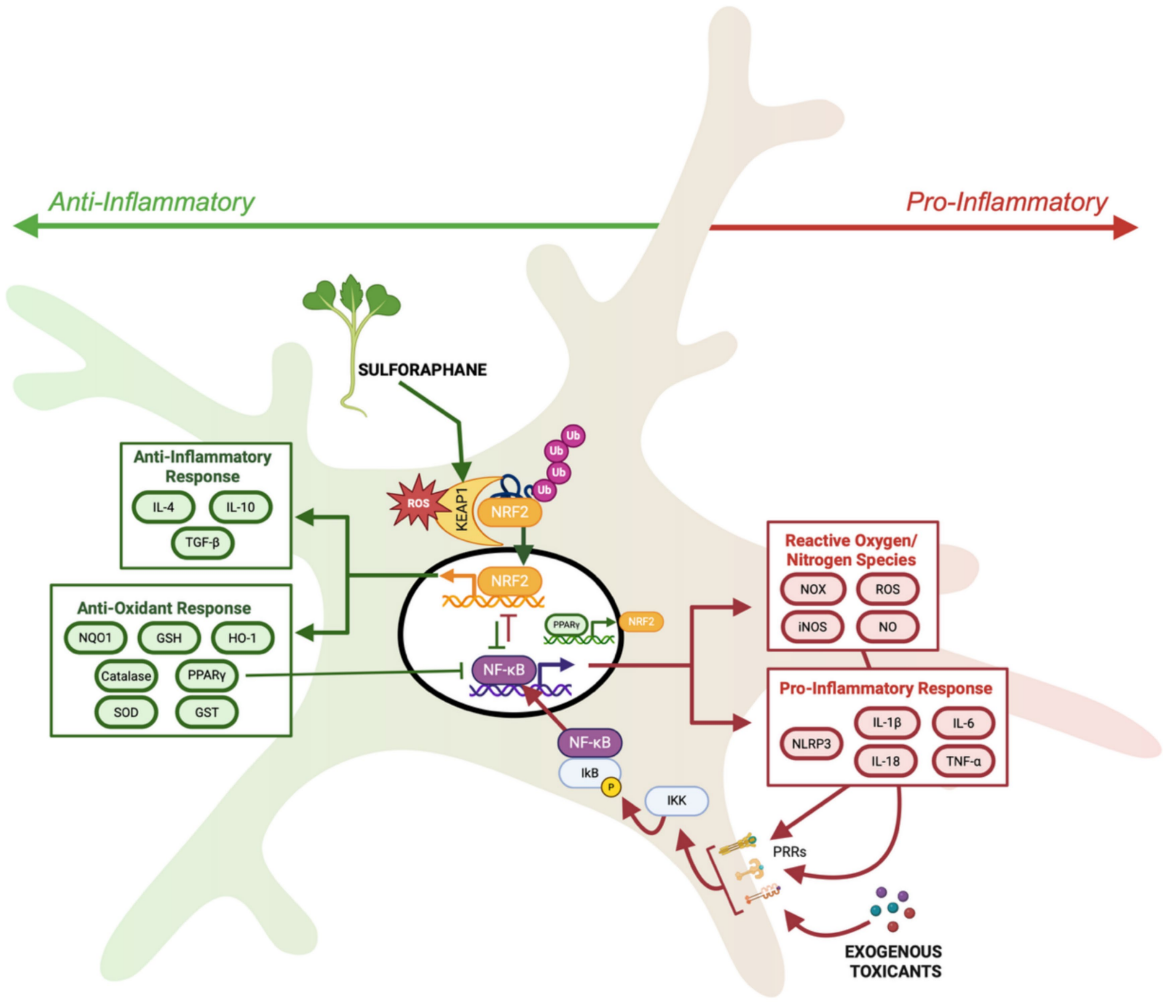
Schematic of NRF2 activation by oxidative stress and sulforaphane through alteration of KEAP-NRF2 interaction leading to transcription of anti-oxidant and anti-inflammatory genes, facilitating increased NRF2 activation through reciprocal activation loop with PPARγ, and inhibiting NF-κB mediated transcription. While NRF2 contributes to anti-inflammatory cellular response, NF-κB drives the transcription of pro-inflammatory cytokines and pro-oxidative enzymes to increase pro-inflammatory cellular responses. Created in BioRender under university license (https://BioRender.com/lfgumjv).

In an effort to identify NRF2 structural domains responsive to oxidative stress and electrophilic compounds, such as sulforaphane, researchers uncovered a novel regulator of NRF2 (Itoh et al., 1999). Itoh et al. (1999) identified this novel protein, Kelch-like ECH-associated protein1 (KEAP1), as the cytosolic negative regulator of NRF2 activity. Future studies demonstrated that KEAP1 does not passively sequester NRF2 in the cytosol, but actively functions as a ubiquitin ligase, leading to ubiquitination and proteasomal degradation of NRF2 (Zhang and Hannink, 2003). Importantly, it is the NRF2-KEAP1 interaction that creates a sensitive intracellular sensor system for oxidative stress, that can also be manipulated by electrophiles. This is possible due to cysteine residues present on KEAP1 that can be oxidized and prevent the otherwise inevitable ubiquitination and degradation of NRF2. In response to oxidative stress, this regulatory mechanism allows NRF2 to rapidly translocate to the nucleus and activate transcription of antioxidant and phase II detoxifying enzymes to effectively mitigate external insults (Figure 2).

Interestingly, many of the initial studies elucidating NRF2-ARE-mediated cellular protection utilized *tert*-butyl hydroquinone (tBHQ), a synthetic antioxidant and inducer of the NRF2 pathway via its auto-oxidized, electrophilic form tert-butyl benzoquinone (tBQ) (Rushmore and Pickett, 1990; Dhakshinamoorthy and Jaiswal, 2001; Abiko et al., 2011). Notably, NRF2-inducing compounds, such as tBHQ and sulforaphane, modify distinct KEAP1 residues from oxidation by ROS, thus allowing for an additive effect in the presence of oxidative stress (Suzuki et al., 2019; Suzuki et al., 2023; Li and Kong, 2009). In many cases, this additive effect is necessary for cellular protection. For example, while glutamate excitotoxicity increases oxidative stress and activates NRF2 signaling, it is often insufficient to prevent cell death (Xin et al., 2019; Habas et al., 2013). However, early tBHQ research demonstrated that the addition of tBHQ increases NRF2-mediated transcription and confers neuroprotection from glutamate-induced oxidative damage (Shih et al., 2003; Kraft et al., 2004). However, despite these promising effects, reports regarding tBHQ’s genotoxicity (Eskandani et al., 2014), pro-oxidative behavior (Imhoff and Hansen, 2010), and potential carcinogenicity (Gharavi et al., 2007) diminished its therapeutic appeal. Thus, sulforaphane gained traction as a safer NRF2 activator, outpacing tBHQ in research focused on neuroprotection.

This review explores the neuroprotective mechanisms of sulforaphane, focusing on its role in mitigating oxidative stress and regulating neuroinflammation. Given its ability to activate NRF2-mediated cytoprotective mechanisms, sulforaphane has emerged as a promising candidate for therapeutic intervention in neurodegenerative and neurodevelopmental disorders. By examining current evidence from cellular, animal, and clinical studies, this review aims to provide a detailed evaluation of sulforaphane’s potential as a neuroprotective agent in neurological disease treatment and prevention.

## 3 Mechanisms of sulforaphane-based neuroprotection

### 3.1 Sulforaphane and oxidative stress regulation

Reactive oxygen species (ROS), such as superoxide anions and hydrogen peroxide, are byproducts of cellular metabolism and function within organelles such as mitochondria, endoplasmic reticulum, and peroxisomes (Juan et al., 2021). While ROS play a role in cellular signaling and homeostasis at low levels, excess ROS production and/or insufficient antioxidant scavenging leads to oxidative stress (Oswald et al., 2018; Averill-Bates, 2024; Kurutas, 2016). Acutely, excess ROS engages cellular defenses, such as the NRF2 pathway and integrated stress response, allowing the cell to divert resources to mitigating oxidative stress. However, prolonged consequences of oxidative stress include the modification of proteins, lipids and nucleic acids, impairing cellular function. These consequences are particularly detrimental within neurons due to their high metabolic demand and limited regenerative capacity (Cobley et al., 2018). While many neurological conditions exhibit distinct pathologies, oxidative stress is a common factor in their etiology. For example, oxidative stress is associated with numerous neurodevelopmental disorders, including Down syndrome, Rett syndrome, Fragile X syndrome, and infantile epilepsy (Buczyńska et al., 2023; De Felice et al., 2012; Pagano et al., 2024; Aguiar et al., 2012). Not surprisingly, many of these syndromic disorders/epilepsies are co-morbid with autism spectrum disorders (ASD), where oxidative stress is a major driver of early synaptic alterations and neuroinflammation (Aguiar et al., 2012; Liu et al., 2022; Bjørklund et al., 2020). In addition to these neurodevelopmental disorders arising from alterations in early brain development, oxidative stress also contributes to psychoses, such as schizophrenia (Bošković et al., 2011), and neurodegenerative diseases, such as Alzheimer’s Disease and Parkinson’s Disease (Gella and Durany, 2009; Cheignon et al., 2017; Dias et al., 2013). Furthermore, oxidative stress in early development may contribute to the increased risk of neurodegeneration later in life, thus linking neurodevelopmental and neurodegenerative disorders (Litwa, 2022).

As described above, sulforaphane modifies cysteine residues within the KEAP1 ubiquitin ligase, albeit distinct cysteine residues from those that become oxidized by ROS (Suzuki et al., 2019). This allows oxidative stress and sulforaphane to have an additive effect on NRF2 activation. Sulforaphane-mediated KEAP1 modification releases NRF2 from degradation and results in NRF2 phosphorylation and nuclear translocation (Figure 2). Within the nucleus, NRF2 binds to promoter regions of target genes which contain antioxidant response element (ARE) sequences, ultimately inducing the transcription of cytoprotective genes, i.e., those encoding for phase II detoxification enzymes and antioxidants. For example, studies using rodents and primary neuron cultures have demonstrated that sulforaphane upregulates key antioxidant enzymes, such as glutathione-S-transferase (GST), NAD(P)H oxidoreductase 1 (NQO1), and heme oxygenase-1 (HO-1), leading to reduced oxidative burden in both neurodevelopmental and neurodegenerative models (Innamorato et al., 2008; Vauzour et al., 2010; Jazwa et al., 2011; Morroni et al., 2013; Tian et al., 2019; Nadeem et al., 2019; Sandouka and Shekh-Ahmad, 2021; Li et al., 2022). Importantly, the majority of sulforaphane’s neuroprotective effects are mediated by NRF2, since the absence of NRF2 prevents sulforaphane’s effects (Innamorato et al., 2008; Jazwa et al., 2011; Li et al., 2022).

### 3.2 Sulforaphane’s role in mitigating neuroinflammation

Neuroinflammation is defined as an immune response within the central nervous system (CNS), characterized by the activation of microglia and astrocytes, increased pro-inflammatory cytokines, and potential disruption of neuronal homeostasis (DiSabato et al., 2016). While neuroinflammation is an innate mechanism designed to protect against infections and injuries, chronic or dysregulated neuroinflammation contributes to the development and progression of CNS disorders ranging from early developmental disorders such as autism spectrum disorders (ASD) (Han et al., 2021) and schizophrenia (Müller et al., 2015) to neurogenerative disorders (Zhang et al., 2023) including Alzheimer’s and Parkinson’s disease (Gella and Durany, 2009; Cheignon et al., 2017; Dias et al., 2013). Often exacerbated by oxidative stress, the CNS resident glia sense and respond to their environment. The resident immune cells of the CNS, microglia, are key players in homeostatic maintenance, response to injury, and mediating neuroinflammatory processes. Microglia populate the CNS during early embryogenesis and primarily function in the phagocytosis of cellular debris, synaptic pruning during neurodevelopment and maturation, and the release of cytokines and growth factors that influence neuronal survival and plasticity (Ginhoux and Prinz, 2015). Under physiologic conditions, microglia exhibit a ramified morphology that enables them to survey their environment (Woodburn et al., 2021). However, pathologic conditions activate microglia to increase cytokine production, resulting in either a pro-inflammatory or anti-inflammatory phenotype, classically defined as M1 and M2, respectively, (Woodburn et al., 2021; Jurga et al., 2020; Gao et al., 2023; Qin et al., 2023; Darwish et al., 2023). Additionally, microglial activation is associated with a morphologic transition from ramified to amoeboid-like, adopting a phagocytic phenotype (Woodburn et al., 2021). This tightly regulated process begins with signal detection by microglia through pattern recognition receptors (PRRs), such as toll-like receptors (TLRs), or other damage associated receptors, such as receptor for advanced glycation end products (RAGE) and scavenger receptors, prompting a transition from a surveillant to a reactive state (Kraft and Harry, 2011). The M1 phenotype is often associated with disease, resulting in production and secretion of pro-inflammatory cytokines (i.e., tumor necrosis factor-alpha (TNFα), interleukin-1*β* (IL-1β), and IL-6), chemokines, and ROS to neutralize pathogens and increase cell recruitment to the site of inflammation. Conversely, the M2 phenotype supports tissue repair and synaptic remodeling following the pro-inflammatory phase through the secretion of anti-inflammatory cytokines [i.e., IL-10 and transforming growth factor-beta (TGF-β)].

The canonical nuclear factor kappa-B (NF-κB) pathway in microglia is a critical regulator of inflammation and drives the M1 phenotype (Anilkumar and Wright-Jin, 2024). Key players in this pathway include PRRs which detect external stimuli like bacterial or viral products, inflammatory cytokines, and chemical stressors. TLR4 is one such PRR that detects damage-associated molecular patterns (DAMPs) created by stressed and dying cells and pathogen-associated molecular patterns (PAMPs) like bacterial lipopolysaccharide (LPS) (Guo et al., 2022; Kumar, 2019). Upon detection of external stimuli, adaptor proteins are recruited to PRRs to activate downstream NF-κB signaling. The NF-κB family includes five members, with the RelA/p50 heterodimer driving transcription in the canonical pathway (Oeckinghaus and Ghosh, 2009). In the canonical pathway, inhibitor of κB (IκB) sequesters the RelA/p50 form of NF-κB in the cytosol, thereby preventing nuclear translocation. However, in response to immune activation, IκB kinase (IκK) phosphorylates IκB, releasing NF-κB from subsequent proteasomal degradation. NF-κB then translocates to the nucleus where it binds specific κB sites to induce the transcription of pro-inflammatory genes, including TNFα, IL-1β, and IL-6 (Anilkumar and Wright-Jin, 2024; Guo et al., 2022; Oeckinghaus and Ghosh, 2009). Microglia M1 activation is accompanied by a metabolic shift from oxidative phosphorylation to glycolysis to support the rapid production of pro-inflammatory cytokines (Wilkinson and Landreth, 2006). While presumably such a shift would reduce mitochondrial production of reactive oxygen species, other sources including NADPH oxidase (NOX) (Wilkinson and Landreth, 2006; Simpson and Oliver, 2020; Rojo et al., 2014) and inducible nitric oxide synthase (iNOS) (Rojo et al., 2014) facilitate rapid generation of superoxide radicals and nitric oxide (NO) aiding in pathogen elimination and contributing to neuroinflammation during aberrant activation. Furthermore, pro-inflammatory cytokines can damage mitochondria and aberrantly increase mtROS production (Lin et al., 2022). Both NF-κB signaling and mtROS promote NLPR3 inflammasome assembly, a key driver of neuroinflammation that leads to cleavage of precursor cytokines into biologically active pro-inflammatory cytokines, IL-1β and IL-18 (Blevins et al., 2022). The tight link between oxidative stress and neuroinflammation presents a compelling target for therapeutic use of NRF2 activators like sulforaphane in neuroinflammatory conditions.

In contrast to pro-inflammatory microglial activation, M2 microglia activation increases anti-inflammatory cytokine production, promoting neuroprotection through tissue repair and debris clearance. Many factors modulate the transition from M1 to M2. For example, anti-inflammatory cytokines such as IL-4 and IL-10, promote the transition to M2 microglia (Zuiderwijk-Sick et al., 2021; Lobo-Silva et al., 2016). In the case of IL-4, downstream signaling leads to the activation of the peroxisome proliferator-activated receptor gamma (PPARγ) transcription factor (Guo et al., 2022). PPARγ is a ligand-activated transcription factor that is known to act synergistically with NRF2 by suppressing NF-κB and promoting the transcription of antioxidant genes including GPX-3 and catalase (Villapol, 2018; Lee, 2017). Furthermore, NRF2 and PPARγ engage in a reciprocal activation loop, enhancing each other’s transcription (Lee, 2017). Notably, PPARγ alone is not sufficient to restore redox homeostasis and promote the M2 phenotype, as demonstrated by the exacerbation of neuroinflammatory cytokine production in NRF2-deficient mice challenged with LPS (Innamorato et al., 2008). Oxidative stress serves as a primary activator of the NRF2 pathway, initiating the transcription of antioxidant and detoxification genes that help restore redox balance. This resolution of ROS is crucial, as excess oxidative stress sustains nuclear factor kappa B (NFκB)-driven inflammation and reinforces the M1 phenotype. By counteracting oxidative stress and promoting anti-inflammatory signaling, NRF2 activators like sulforaphane may facilitate the transition to the neuroprotective M2 phenotype.

Accumulating evidence highlights sulforaphane’s influence on microglial activation. Bacterial LPS is a common mechanism to stimulate microglia activation of pro-inflammatory cytokine release. In this model, sulforaphane reduced expression of pro-inflammatory cytokines, while restoring production of anti-inflammatory cytokines to control levels. These changes are accompanied by decreased NFκB signaling and increased NRF2 activation (Innamorato et al., 2008; Subedi et al., 2019; Eren et al., 2018). Other external stressors similarly stimulate pro-inflammatory cytokine production, including chemical, metabolic, physical and social stressors. In these models, sulforaphane promoted resilience by preventing pro-inflammatory cytokine production and associated adverse consequences. For example, in either LPS or chronic social defeat stress (CSDS) models of depression, sulforaphane prevented stress-induced increases in pro-inflammatory cytokine production and restored anti-inflammatory cytokine production (Tang et al., 2022). Furthermore, sulforaphane promoted microglial ramification similar to physiological conditions. These effects were accompanied by increased NRF2-mediated transcription of brain-derived neurotrophic factor (BDNF) and decreased levels of methyl-CpG binding protein 2 (MECP2), a negative regulator of BDNF production (Tang et al., 2022). Through these microglial effects, sulforaphane prevented stress-induced synaptic loss and significantly reduced depressive-like behaviors.

In addition to microglia, astrocytes and peripheral immune cells can also contribute to neuroinflammatory processes. In astrocytes, LPS also increases NFκB-mediated transcription of pro-inflammatory cytokines, including TNFα, IL-1β, iNOS and cyclooxygenase-2 (COX2) (Bobermin et al., 2020). Inflammatory messengers, such as TNFα, can also interact with astrocytes to promote ROS generation and apoptosis (Liu et al., 2020). In both cases, sulforaphane exerts a neuroprotective role, suppressing inflammatory signaling and ROS generation (Bobermin et al., 2020; Liu et al., 2020). Chronic neural inflammation and central nervous system (CNS) injury can also increase blood brain barrier permeability, allowing immune cells such as macrophages to migrate into the CNS from the bloodstream (Minogue, 2017). Similar to microglia and astrocytes, sulforaphane prevents LPS-mediated macrophage activation, inhibiting iNOS expression and NO generation (Heiss et al., 2001). Interestingly, this study shows efficacy for the simultaneous application of sulforaphane but not post-treatment, indicative of time and dose-dependent effects of sulforaphane against toxicants. Thus, sulforaphane’s powerful anti-inflammatory properties extend beyond microglia to promote resiliency of neural networks.

## 4 Beneficial properties of sulforaphane in neuronal disorders

### 4.1 Sulforaphane and neurodevelopmental disorders

#### 4.1.1 Epileptic seizures

Many models have been developed to study seizurogenic activity. For example, in the brain, magnesium (Mg^2+^) exerts anti-seizurogenic properties by antagonizing N-methyl-D-aspartate receptor (NMDAR)-excitatory activity (Ruppersberg et al., 1994). Thus, hypomagnesia can result in seizures in patient populations (Ruppersberg et al., 1994; Michael and George, 2022). Mg^2+^ removal from media can also be used to model seizure-like activity in neuronal cultures. Rapidly, within 10 min of Mg^2+^ removal, ROS production significantly elevates. However, sulforaphane co-administration blunts ROS elevation through increased expression of the antioxidant scavenger, glutathione, thereby preventing neuronal cell death (Sandouka and Shekh-Ahmad, 2021). Importantly, sulforaphane-mediated NRF2 activation can also be neuroprotective in models of induced epilepsy when administered post-seizure. The glutamate analog kainic acid (KA) has been used in many mammals to induce status epilepticus (KA-SE) through its neuroexcitatory and neurotoxic effects (Nadler and Kainic, 1981). In rats, the KA-SE model also exhibits a decrease in reduced glutathione (GSH) and an increase in its oxidized form (GSSG), indicating increased ROS burden post seizure activity. This increase in ROS correlates with an increase in neuronal death in KA-SE rats. Sulforaphane administered as a post-treatment restores depleted GSH levels, increasing antioxidant capacity and preventing cell loss (Sandouka and Shekh-Ahmad, 2021). Thus, sulforaphane either during or following a seizurogenic event can reduce neuronal loss.

Given the central role of oxidative stress in seizure-related neuronal damage, mitochondria emerge as a potential target for oxidative insult. As mitochondria are a central hub for energy production in neurons, they are particularly vulnerable to oxidative damage. Mitochondrial damage and subsequent dysfunction not only reduce energy production but also increases ROS generation, creating a self-perpetuating cycle of oxidative stress and metabolic impairment. Consequently, many metabolic disorders are associated with risk of epilepsy due in part to the brain’s high energy demand and reliance on energy production for homeostasis (Fei et al., 2020). In epilepsy, this interplay is evident in models such as the lithium-pilocarpine (Li-Pilo) model of SE, which recapitulates key features of epileptogenesis including altered glucose metabolism (Daněk et al., 2022) and mitochondrial bioenergetics with increased markers of oxidative damage (Carrasco-Pozo et al., 2015; Folbergrová et al., 2023). In both immature (Carrasco-Pozo et al., 2015; Folbergrová et al., 2023) and adult (Carrasco-Pozo et al., 2015) rodent models of Li-Pilo induced SE, sulforaphane exhibits beneficial effects, reducing oxidative stress-induced damage and improving cellular metabolism. Despite these promising effects of sulforaphane in epilepsy models, to date there are no clinical trials addressing its safety and efficacy in this patient population.

#### 4.1.2 Autism Spectrum disorders

Surprisingly, there are few studies looking at sulforaphane’s effectiveness in autism preclinical models considering most of the clinical trials (discussed later) for sulforaphane in neurological conditions focused on the ASD patient pool. ASD can be modeled in mice through genetic modifications, pharmacological interventions, and immune challenges during pregnancy. One widely used inbred strain—(BTBR T + Itpr3tf/J) BTBR mice—exhibits behavioral phenotypes similar to patients with ASD (Endo et al., 2019). For example, compared to other genetic backgrounds like the C57BL/6 mice, BTBR mice have impaired social interaction and autism-stereotyped repetitive behaviors (Nadeem et al., 2019; Endo et al., 2019). In the cerebellum, which is increasingly recognized for its contributions to ASD core symptoms (D’Mello and Stoodley, 2015), BTBR mice show increased NFκB and iNOS expression as well as lipid peroxides, indicative of increased oxidative stress (Nadeem et al., 2019). Sulforaphane attenuated these components of oxidative stress and neuroinflammation, reducing autism-like behaviors to control levels. Additionally, sulforaphane increased NRF2 target gene expression and enhanced activity of antioxidant enzymes, including superoxide dismutase (SOD), glutathione peroxidase (GPx), and glutathione reductase (GR). These findings demonstrate that sulforaphane robustly activates NRF2 in the cerebellum and peripheral tissues, protecting them from oxidative stress and leading to a reduction in autism-like behaviors (Nadeem et al., 2019). Epidemiological data has linked maternal immune infection (MIA) during pregnancy to risk of ASD development in human offspring (Jones et al., 2017; Brown et al., 2014; Goines et al., 2011). Similarly, MIA can be modeled in rodents using the toll-like receptor 3 agonist polyriboinosinic-polyribocytidilic acid poly(I:C), which leads to autism-like behavior in offspring (Fujita et al., 2020). However, when the precursor to sulforaphane, glucoraphanin, was provided in the mother’s food during pregnancy and lactation, the pups had significant improvement in cognitive deficits and social interaction and protection from poly(I:C) induced parvalbumin-positive cell loss. This finding is particularly interesting because it looks at prenatal exposure outcomes using sulforaphane as a preventative drug rather than a therapeutic to attenuate developed behaviors. Building on these findings, our recent study demonstrated that sulforaphane prevented ASD-associated phenotypes induced by valproic acid, including oxidative stress, synaptic loss and neural activity disruption, highlighting sulforaphane’s potential as a preventative strategy against early life ASD risk (Bessetti et al., 2025).

### 4.2 Sulforaphane and schizophrenia

Schizophrenia is a neurological disorder with complex clinical presentation which includes symptoms like hallucinations and psychosis, as well as other symptoms like social withdrawal and cognitive impairments. While hallucinations and psychosis are inherently subjective and difficult to measure directly in animal models, researchers have developed behavioral assays that can assess features of the disorder (Winship et al., 2019). For example, prepulse inhibition (PPI), a neurological phenomenon where a weaker, non-startling stimulus “prepulse” reduces the response to the subsequent stronger, startling stimulus “pulse” is often reduced in schizophrenia, as well as other neuropsychiatric disorders. As a result, these patients can become easily overwhelmed or distracted, due to the inability of their CNS to filter out irrelevant information - this process is known as sensorimotor gating (Winship et al., 2019). In addition to PPI, social interaction tests and cognitive tests can be used as measurable behavioral and neurological outcomes of disease models. Similar to epilepsy models, rodent models of schizophrenia often employ pharmacological treatments to induce schizophrenia-like phenotypes. For example, the NMDAR antagonist phencyclidine (PCP) can induce hyperlocomotion and PPI deficits (Shirai et al., 2012; Swerdlow et al., 2016), as well as cognitive impairment (Shirai et al., 2015) in rodent models, recapitulating key characteristics of schizophrenia. In this model, sulforaphane administered at 30 mg/kg can attenuate PCP-induced hyperlocomotion and PPI deficits (Shirai et al., 2012). Moreover, sulforaphane rescues PCP-induced cognitive defects and reductions in dendritic spine density (Shirai et al., 2015). Animals exposed to PCP treatment increased 8-oxo-dG, indicative of DNA/RNA damage from oxidative stress, after repeat administration. This oxidative stress, likely driven by altered neurotransmitter signaling and cellular metabolism, is thought to contribute to schizophrenia-related deficits in inhibitory interneuron populations, including parvalbumin-positive cells (Li et al., 2024). Sulforaphane pre-treatment protects from oxidative damage, reducing 8-oxo-dG positive cells to the level observed in control animals and preventing loss of parvalbumin-positive cells, thereby preventing schizophrenia-like behaviors (Shirai et al., 2015).

### 4.3 Sulforaphane in neurodegenerative diseases

#### 4.3.1 Alzheimer’s disease

The ability of sulforaphane to counteract oxidative stress is not confined to early brain development and adolescence; studies have also highlighted its therapeutic potential in neurodegenerative disorders where chronic oxidative damage contributes to neuronal dysfunction. For instance, in Alzheimer’s disease (AD) models, sulforaphane administration enhanced antioxidant defense, reduced oxidative damage and improved behavioral outcomes. The accumulation of amyloid-*β* (Aβ) is a pathological hallmark of AD, with Aβ being produced through the cleavage of amyloid precursor protein (APP) by β-and *γ*-secretases, generating peptides of Aβ of varying length (Murphy and LeVine, 2010). The longer forms of Aβ result in a more hydrophobic and fibrillogenic peptide. The 42 amino acid peptides readily form oligomers that can further accumulate into fibrils and plaques in the brain, which are hallmarks of AD pathology (Cheignon et al., 2017; Murphy and LeVine, 2010). While the exact mechanism and involvement of oxidative stress in AD pathogenesis have yet to be elucidated, elevated levels of Aβ and increased byproducts of cellular oxidation to lipids, proteins and nucleic acid are closely associated in patient populations (Gella and Durany, 2009; Cheignon et al., 2017; Chang et al., 2014) and are recapitulated in mouse models (Cheignon et al., 2017). Indeed, transgenic mice given the human presenilin-1 (PS1) gene—which encodes one of the two subunits of *γ*-secretase—with one missense mutation (Val97Leu, PS1V97L) formed abundant Aβ oligomers (Zhang et al., 2014), produced cognitive deficits, and exhibited cellular oxidative stress markers (Tian et al., 2019; Hou et al., 2018). Sulforaphane supplementation decreased amyloid plaque burden and improved cognitive performance in PS1V97L transgenic mice. This effect correlated with increased NRF2 activation and upregulated mitochondrial antioxidant enzymes (Tian et al., 2019; Hou et al., 2018). Aβ oligomer toxicity studies in primary cortical neuron cultures further supported these findings. Sulforaphane pre-treatment (0.1 μM) increased cell viability and protected from Aβ oligomer-induced dendrite loss (Hou et al., 2018).

These amyloid oligomers also contribute to the neuroinflammatory signatures of neurodegenerative diseases. Amyloid oligomers and plaques are one of many DAMPs that activate microglia M1 phenotypes. Downstream DAMP-associated microglia activation results in the assembly of the classical NADPH oxidase on the plasma membrane, resulting in the release of superoxides into the extracellular space, where they contribute to neuronal damage (Della Bianca et al., 1999). Furthermore, PS1V97L transgenic mice exhibited increased inflammatory cytokines including IL-1β and TNFα, which were attenuated by sulforaphane supplementation (Hou et al., 2018). In many experimental models, Aβ is studied in isolation due to its prominent role in AD pathogenesis. However, Aβ toxicity is mechanistically linked to tau pathology, particularly tau hyper-phosphorylation and aggregation, which lead to neurofibrillary tangle (NFT) formation (Bloom, 2014). Thus, tau hyper-phosphorylation is another neuropathologic hallmark of AD and is linked to microglia activation and neuronal damage (Medeiros et al., 2010; Grundke-Iqbal et al., 1986; Jiang et al., 2021). Tau pathology is strongly associated with both AD and broader dementia phenotypes (Medeiros et al., 2010). Hyper-phosphorylation of tau and NFTs correlated more directly with the progression of cognitive impairment and disease severity than Aβ burden, making it a critical therapeutic target (Huber et al., 2018; Arriagada et al., 1992). Importantly, sulforaphane demonstrated therapeutic efficacy in preclinical models involving pathogenic tau. Sulforaphane suppressed tau hyper-phosphorylation and improved cognitive deficits in mouse models of AD, as well as in models of dementia-associated vascular cognitive impairment and metabolic dysfunction-driven diabetes mellitus (Hou et al., 2018; Li et al., 2025; Lee et al., 2018; Pu et al., 2018). Collectively, these findings highlight sulforaphane’s neuroprotective potential to counteract both Aβ and tau-related neurodegenerative processes in AD and other dementias.

#### 4.3.2 Parkinson’s disease

Oxidative stress has also been linked to the degeneration of dopaminergic neurons in Parkinson’s Disease (PD) (Dias et al., 2013). Current data suggests that dopamine metabolism, low glutathione (GSH) and high levels of iron and calcium contribute to ROS accumulation in dopaminergic neurons (Dias et al., 2013). Preclinical studies have used various neurotoxin-based models to mimic the oxidative stress and dopaminergic neurodegeneration observed in PD. The 1-methyl-4-phenyl-1,2,3,6-tetrahydropyridine (MPTP), 6-hydroxydopamine (6-OHDA), and 5-S-cysteinyl-dopamine (CysDA) models each target dopaminergic neurons and increase oxidative damage to the brain, providing valuable insight to the efficacy of mitigating oxidative stress as a mechanism of sulforaphane’s neuroprotective effects. Studies using MPTP-induced Parkinson’s models demonstrated sulforaphane treatment preserved tyrosine hydroxylase-positive (dopaminergic) neurons (Jazwa et al., 2011; Galuppo et al., 2013), and reduced motor impairments (Galuppo et al., 2013). This neuroprotection was attributed to sulforaphane’s NRF2 activation and upregulation of phase II antioxidant response. Evaluation of the brain tissue using immunohistochemistry and immunofluorescence showed an increase in both GFAP and Iba-1 positive cells in wild type mice treated with MPTP. The MPTP-induced astrogliosis and microgliosis, respectively, was reduced by sulforaphane co-treatment with MPTP injection at both three days and six days post treatment. These results correlated with reduced inflammatory cytokines IL-6 and TNFα, upregulation of phase II enzymes associated with co-treatment with sulforaphane (Jazwa et al., 2011). Similarly, in the 6-OHDA model, sulforaphane prevented degeneration of dopaminergic neurons and improved rotarod latency to fall behavior (Morroni et al., 2013). Sulforaphane effectively prevented 6-OHDA-induced DNA fragmentation, activation of caspase-3 and depletion of the glutathione redox system (GSH, GST, and GR) in brain tissues (Morroni et al., 2013). In primary cortical neurons, exposure to 5-S-cysteinyl-dopamine (CysDA), a neurotoxic derivative of dopamine oxidation, results in a dose dependent reduction in cell survival. Sulforaphane (0.1 μM) was shown in this model to be protective against CysDA-induced neuronal injury, correlating with increased NRF2 pathway activation, enhanced NQO1 activity and restored GSH levels (Vauzour et al., 2010). Together these studies provide compelling evidence for sulforaphane—through NRF2-mediated cytoprotective pathways—to reduce oxidative stress, neural inflammation and cell death in chemically-induced models of PD.

Beyond oxidative damage, alpha-synuclein (*α*-syn) aggregation is a central pathological hallmark of PD, as it is the major component of Lewy bodies—abnormal deposits of proteins found in the brains of patients with PD (Paulėkas et al., 2024). The accumulation and misfolding of *α*-syn is due in part to post-translation modification, particularly phosphorylation and nitration that can be exacerbated by oxidative stress (Brembati et al., 2023). While the chemically induced models of PD, MPTP and 6-OHDA, provide valuable insight into the oxidative stress-related mechanisms of dopaminergic neuron loss *in vivo*, they do not fully recapitulate PD neuropathology with *α*-syn aggregation (Meredith and Rademacher, 2011; Cui et al., 2023). This further supports the notion that the interplay between oxidative stress and a-syn is not fully understood (Brembati et al., 2023). However, sulforaphane—through its well-characterized activation of the NRF2 pathway and enhancement of cellular antioxidant responses—may reduce oxidative post-translational modifications that facilitate α-syn aggregation. This highlights the need for further research on sulforaphane’s efficacy in models that more directly recapitulate Lewy body formation and α-synucleopathies.

### 4.4 Summary of sulforaphane in preclinical models of neuronal disorders

Preclinical sulforaphane studies in cell culture highlight an emerging theme of sulforaphane-mediated neuroprotection, namely the efficacy of low sulforaphane doses (Table 2). In neuronal cell culture models, sulforaphane consistently demonstrates neuroprotection as low as 0.01 μM (Vauzour et al., 2010). with many studies showing efficacy at 0.1 μM (Vauzour et al., 2010; Tian et al., 2019; Bessetti et al., 2025; Hou et al., 2018). Notably, in a CysDA model of PD, sulforaphane-mediated improvement in neuronal viability peaked at 0.1 μM and had no benefit at 10 μΜ (Vauzour et al., 2010). Similarly, we observed that 0.1 μM sulforaphane, but not 1 or 10 μM, increased NRF2 nuclear translocation in neural progenitor cells (Bessetti et al., 2025). While other brain cell types, such as astrocytes and microglia, may be responsive to slightly higher doses, they too benefit from lower sulforaphane doses (Subedi et al., 2019; Eren et al., 2018; Tang et al., 2022; Bobermin et al., 2020; Liu et al., 2020; Heiss et al., 2001). For example, sulforaphane significantly decreased microglial cell viability at 50 μM (Eren et al., 2018). However, translation of sulforaphane doses to *in vivo* preclinical animals is complicated by tissue bioavailability and metabolic differences. Most animal models exhibit neuroprotective benefits between 5 mg/kg up to 50 mg/kg (Table 2). However, notably in a PCP model of schizophrenia, only 30 mg/kg, but not 3 or 10 mg/kg, rescued defects in hyperlocomotion and PPI, in stark contrast to cell culture models where lower models were more beneficial (Shirai et al., 2012). This could be due to the rapid metabolism and excretion of sulforaphane *in vivo* (Fahey et al., 2015). In contrast, the precursor, glucoraphanin, is much more stable and exhibits higher bioavailability, particularly when administered with active myrosinase (Fahey et al., 2015). Thus, in animal models, we observe increasing delivery of bioactive sulforaphane through its precursor glucoraphanin, usually at 0.1% of food content (Table 2). We will continue to see this trend in administration of glucoraphanin in clinical trials discussed below.

**Table 2:** Preclinical sulforaphane studies.

*In vitro* models					
Cell type	SFN or precursor	Dose(s)	Insult	Primary outcomes/findings	Citation
BV2 immortalized mouse microglia cell line and RAW264.7 murine macrophages	SFN	**1 μM, 5 μM, and 10 μM**	100 ng/mL LPS	⇑ NRF2 target gene HO-1, NRF2 protein, and anti-inflammatory cytokines ⇓ LPS-induced pro-inflammatory mediators and cytokines	Subedi et al. (2019)
N9 microglial cells, SH-SY5Y cells, and mouse primary microglia (PND 2)	SFN	**1 μM, 2 μM, 5 μM, 10 μM**, 20 μM, and 50 μM	100 ng/mL LPS and NRF2 siRNA	⇓ Cell viability ≥20μM SFN ⇑ Cell viability of LPS exposed cells with 1, 2, and 5 μM SFN ⇓ LPS-induced pro-inflammatory cytokines, mediators, and ROS ⇑ NRF2 target genes NRF2 siRNA prevents SFN-mediated reduction in pro-inflammatory cytokines	Eren et al. (2018)
BV2 microglial cells	SFN	Not stated	1 μg/mL LPS	⇑ BDNF and NRF2 ⇓ MeCP2	Tang et al. (2022)
C6 astrocyte-like cell line	SFN	1 μM, **5 μM**, and 10 μM	10 μg/mL LPS	⇓ LPS-induced pro-inflammatory mediator and cytokine mRNA expression, extracellular pro-inflammatory cytokines and nuclear NFκB ⇑ NRF2 target gene HO-1 mRNA expression, extracellular anti-inflammatory cytokines, and NRF2 target gene activity (GCL) and content (GSH)	Bobermin et al. (2020)
Pig primary endothelial cells and cortical astrocytes (PND 1–5)	SFN	**1 μM and 5 μM**	TNF-*α* (30 ng/mL) or glutamate (2 mM)	⇓ TNF-α and glutamate-induced ROS, NADPH oxidase activity, and apoptosis	Liu et al. (2020)
RAW 264.7 murine macrophages	SFN	**0.4, 0.8, 1.5 3, 5, 6, 10, 12, 20 and 25 μM**	500 ng/mL LPS	⇓ LPS-induced NO_2_ and TNFα release, iNOS, and Cox-2 expression	Heiss et al. (2001)
Primary Sprague Dawley rat mixed cortical neuron and glial cultures (PND 0–1)	SFN	**5 μM**	Epilepsy model: Mg^2+^ removal	⇓ low Mg^2+^-induced ROS production and epileptiform activity-induced neuronal death ⇑ GSH	Sandouka and Shekh-Ahmad (2021)
Primary E15-16 mouse cortical neurons from AD model PS1V97L-Tg mice and age-matched wild-type C57BL/6	SFN	**0.1 μM**	Genetic AD model NRF2 siRNA	⇓ oxidative stress, Aβ accumulation in a NRF2-dependent fashion ⇑ PS1V97L-Tg neuron viability in a NRF2-dependent fashion	Tian et al. (2019)
Human IPSC derived neural progenitor cells	SFN	**0.1 μM**, 1.0 μM and 10 μM	500 μM Valproic Acid	⇑ NRF2 nuclear localization, transcription of NRF2-ARE mediated genes (SOD, GPX and PPARγ) ⇓ VPA-induced oxidative stress and teratogen-associated gene signatures	Bessetti et al. (2025)
Human IPSC derived cortical spheroids	SFN	**0.1 μM**		⇑ NRF2 nuclear localization ⇓ VPA-induced synaptic alterations	
Primary E18.5 CD-1 mouse cortical neurons				⇓ VPA-induced synaptic alterations to structure and function	
Primary E18 rat cortical neurons	SFN	**0.1 μM**	5, 10, and 20 μM natural Aβ oligomers	⇓ Aβ neurotoxicity and hyper-phosphorylated tau	Hou et al. (2018)
Primary E15 mouse neurons from 3x Tg-AD and non-transgenic mice	SFN	**10 μM**	Genetic AD model with CHIP siRNA	⇓ Aβ and Tau accumulation in a CHIP and HSP70-dependent fashion *Impact of dose on control neuron viability not tested	Lee et al. (2018)
Primary cortical neurons	SFN	**0.01, 0.05, 0.1, 0.3, 0.5, 1, 3, 5, and 10 μM**	100 μM CysDA PD Model	⇓ CysDA-induced toxicity with a hormetic dose response and highest protective effect at 0.1 μM SFN ⇑ NRF2 target genes (GR, GST, TR, and NQO1)	Vauzour et al. (2010)

*In vivo* models					
Animal	SFN or precursor	Dose	Insult	Primary outcomes/findings	Citation
Male adult C57BL/6 mice (8 weeks old), CD1 mice (14 weeks old), and male adult Thy1-Yellow fluorescent protein (YFP) mice	SFN	10 mg/kg i.p.	Social defeat stress for 30 min over 10 days	⇓ Stress-induced pro-inflammatory cytokines and mediators, IBA1 positive immunoreactive microglia, and MeCP2 expression in microglia ⇑ Anti-inflammatory cytokines and NRF2 expression Prevents social interaction deficits and stress-induced dendritic spine density reductions	Tang et al. (2022)
10-day old male Wistar rats	SFN	5 mg/kg i.p.	Status epilepticus: Lithium chloride (Li-Cl) pilocarpine	⇑ Mitochondrial complex IV and V, NFR2 and SOD protein ⇑ glucose uptake and blood flow following SE	Daněk et al. (2022)
Adult male CD1 mice	SFN	5 mg/kg/day i.p. 5 days prior to onset of seizure activity	Status epilepticus: 6 Hz, Li-Cl Pilocarpine, Fluorothyl, and Pentylenetetrazole	Sulforaphane has anticonvulsant effects in multiple seizure models	Carrasco-Pozo et al. (2015)
			Li-Cl pilocarpine	⇑ SOD and CAT activity and improves mitochondrial bioenergetics ⇓ lipid peroxidation and oxidative stress	
12-day old male Wistar rats	SFN	5 mg/kg 48 and 24 h prior to SE induction	status epilepticus: Li-Cl pilocarpine	⇑ Complex I activity ⇓ Li-Cl pilocarpine-induced superoxide production and oxidative damage (3-NT and 4-HNE)	Folbergrová et al. (2023)
Pregnant ddY mice	Glucoraphanin (GF)	0.1% GF food tablets throughout pregnancy and lactation	poly(I: C) maternal immune activation	⇓ poly(I:C)-induced social and cognitive deficits	Fujita et al. (2020)
Male BTBR T + Itpr3tf/J (BTBR) and C57BL/6 (C57) mice (8–10 weeks old)	SFN	50 mg/kg, i.p. per day for 7 days	genetic ASD model	⇓ ASD-like behavior, T-cell activation, and inflammatory mediators in BTBR mice ⇑ NRF2 target gene expression and activity (SOD1, GPx1 and GR)	Nadeem et al. (2019)
Male and Female Sprague–Dawley rats	SFN	5 mg/kg i.p. once daily for 5 days	kainic acid-induced status epilepticus (KA-SE)	⇓ KA-SE-induced neuronal death ⇑ NRF2, NRF2 target gene expression (NQO1 and HO-1), and antioxidant capacity Restored glutathione oxidation/reduction ratios	Sandouka and Shekh-Ahmad (2021)
Male Std:ddy mice (6 weeks old)	SFN	3.0, 10, or 30 mg/kg i.p. prior to PCP injection	Schizophrenia model: PCP (3.0 mg/kg) s.c.	⇓ PCP-induced hyperlocomotion and PPI	Shirai et al. (2012)
Male ICR mice (6 weeks old)	SFN	30 mg/kg/day, i.p.	Schizophrenia model: PCP 10 mg/kg/day s.c.	⇓ PCP-induced cognitive deficits and corresponding spine density loss and oxidative stress marker (8-oxo-dG) in mPFC and CA1	Shirai et al. (2015)
Male ICR mice (4 weeks old)	Glucoraphanin	0.1% glucoraphanin in food			
10-month-old male and female PS1V97L-Tg mice and age-matched wild-type C57BL/6 mice	SFN	5 mg/kg i.p.	Genetic AD model	⇑ Nrf2 expression, NRF2 target gene expression (NQO1, HMOX1, SOD, CAT), improves cognitive function ⇓ oxidative stress and Aβ accumulation	Tian et al. (2019)
Six-month-old male and female PS1V97L Tg mice and aged matched C57BL/6 J WT mice	SFN	5mg/kg i.p daily	Genetic AD model	⇓ Cognitive deficits, Aβ oligomer formation, oxidative stress, and inflammatory cytokines (IL-1β and TNFα) in PS1V97L-TG mice ⇑ GSH	Hou et al. (2018)
Male Sprague–Dawley (SD) rats weighing 300-330 g (6–7 months)	SFN	10 mg/kg i.p. immediately following surgery and twice a week	Chronic hypoperfusion model of vascular cognitive impairment	⇑ NRF2 target gene expression (HO-1), cognitive function after hypoperfusion, and neuronal viability ⇓ hypoperfusion-induced p-Tau and Aβ accumulation	Li et al. (2025)
female 12-month-old 3 × Tg-AD mice and non-transgenic mice	SFN	10 mg/kg/day or 50 mg/kg/day by oral gavage 6 days per week for 8 weeks	Genetic AD model	⇓ Aβ, APP, Tau and p-Tau expression in 3 x Tg-AD mice ⇑ CHIP and HSP70 which are necessary to clear Aβ and Tau SFN prevents learning and memory deficits	Lee et al. (2018)
21 week old male db/db mice (BKS. Cg-Dock7 m +/+ Lepr db /J; genotype: Lep db /Lepr db) and age-matched non-diabetic db/+ control mice (C57BLKS/J)	SFN	1 mg/kg per day for 28 days	Genetic diabetic model with AD-like lesions	⇑ Nuclear NRF2 accumulation and NRF2 target gene expression (HO-1 and NQO1) ⇓ cognitive impairments, Aβ plaque accumulation, p-Tau, and ROS/RNS formation in db/db mice	Pu et al. (2018)
Nrf2-knockout mice and their wild-type littermates	SFN	50 mg/kg i.p. pre-treated twice and three timepoints following MPTP	PD model: MPTP (30 mg/kg) administered every day for 5 days	Sulforaphane crosses the blood brain barrier and is detected in neuronal tissue after i.p. injection ⇑ NRF2 target gene expression (HO-1 and NQO1) ⇓ MPTP-induced dopaminergic neuron loss, neurotoxicity, astrogliosis, microgliosis, and corresponding increase in inflammatory cytokines (IL-6 and TNFα) NRF2-KO prevents NRF2 target gene expression in response to SFN and inhibits protective effects	Jazwa et al. (2011)
Male C57BL/6 mice (4–5 weeks old)	Bioactivated RS-(−)-glucoraphanin	10 mg/kg	PD model: MPTP two injections of 40 mg/kg for acute Parkinsonian syndrome or five injections of 20 mg/kg for sub-acute symptoms	⇓ MPTP-induced dendritic spine and dopaminergic neuron loss, pro-inflammatory cytokine production (IL-1β), oxidative damage (Nitrotyrosine), GFAP positive cells, and pro-apoptotic gene expression (Bax) ⇑ NRF2 expression, and anti-apoptotic gene expression (Bcl2)	Galuppo et al. (2013)
Male C57Bl/6 (9 weeks old)	SFN	5 mg/kg i.p. twice weekly one hour after 6-OHDA injection	PD model: 6-OHDA unilateral stereotaxic intrastriatal injection to the left side	⇓ 6-OHDA-induced motor deficits, DNA damage, and apoptosis ⇑ Cell survival of dopaminergic neurons and proteins involved in glutathione metabolism (GSH, GST and GR)	Morroni et al. (2013)

Studies categorized by in *vitro* and *in vivo* models with SFN or precursor dose used against insult in any assay in bold. 3-NT, 3-Nitrotyrosine; 4-HNE, 4-Hydroxynonenal; 6-OHDA, 6-hydroxydopamine; 8-oxo-dG, 8-Oxo-2′-deoxyguanosine; AD, Alzheimer’s disease; APP, Amyloid Precursor Protein; ARE, Antioxidant Response Element; ASD, Autism Spectrum Disorder; Aβ, Amyloid-beta; BDNF, Brain Derived Neurotropic Factor; CAT, Catalase; CHIP, C-terminus of HSP70-interacting protein; Cox-2, Cyclooxygenase-2; CysDA, 5-S-cysteinyl-dopamine; GCL, Glutamate-Cysteine Ligase; GPx, Glutathione Peroxidase; GR, Glutathione Reductase; GSH, Glutathione; GST, Glutathione-S-Transferase; HMOX1, Heme Oxygenase 1; HO-1, Heme Oxygenase-1; HSP70, Heat Shock Protein 70; i.p., Intraperitoneal; IBA1, Ionized Calcium Binding Adaptor Molecule 1; IL-1β, Interleukin 1-beta; IL-6, Interleukin 6; iNOS, Inducible Nitric Oxide Synthase; IPSC, Induced Pluripotent Stem Cell; KA-SE, Kainic Acid-induced Status Epilepticus; KO, Knockout; Li-Cl, Lithium chloride; LPS, Lipopolysaccharide; MeCP2, Methyl-CpG Binding Protein 2; mPFC, Medial Prefrontal Cortex; MPTP, 1-methyl-4-phenyl-1,2,3,6-tetrahydropyridine; NADPH, Nicotinamide Adenine Dinucleotide Phosphate; NFκB, Nuclear Factor kappa B; NQO1, NAD(P)H Oxidoreductase 1; NRF2, Nuclear Factor Erythroid 2-related Factor 2; PD, Parkinson’s Disease; PCP, Phencyclidine; poly(I, C), Polyinosinic:polycytidylic acid; PPARγ, Peroxisome Proliferator-Activated Receptor Gamma; PPI, Prepulse Inhibition; RNS, Reactive Nitrogen Species; ROS, Reactive Oxygen Species; siRNA, small interfering RNA; s.c., Subcutanious; SE, Status Epilepticus; SFN, Sulforaphane; SOD, Superoxide Dismutase; TNF-α, Tumor Necrosis Factor-alpha; TR, Thioredoxin Reductase.

## 5 Therapeutic implications of sulforaphane

**Figure 3:**
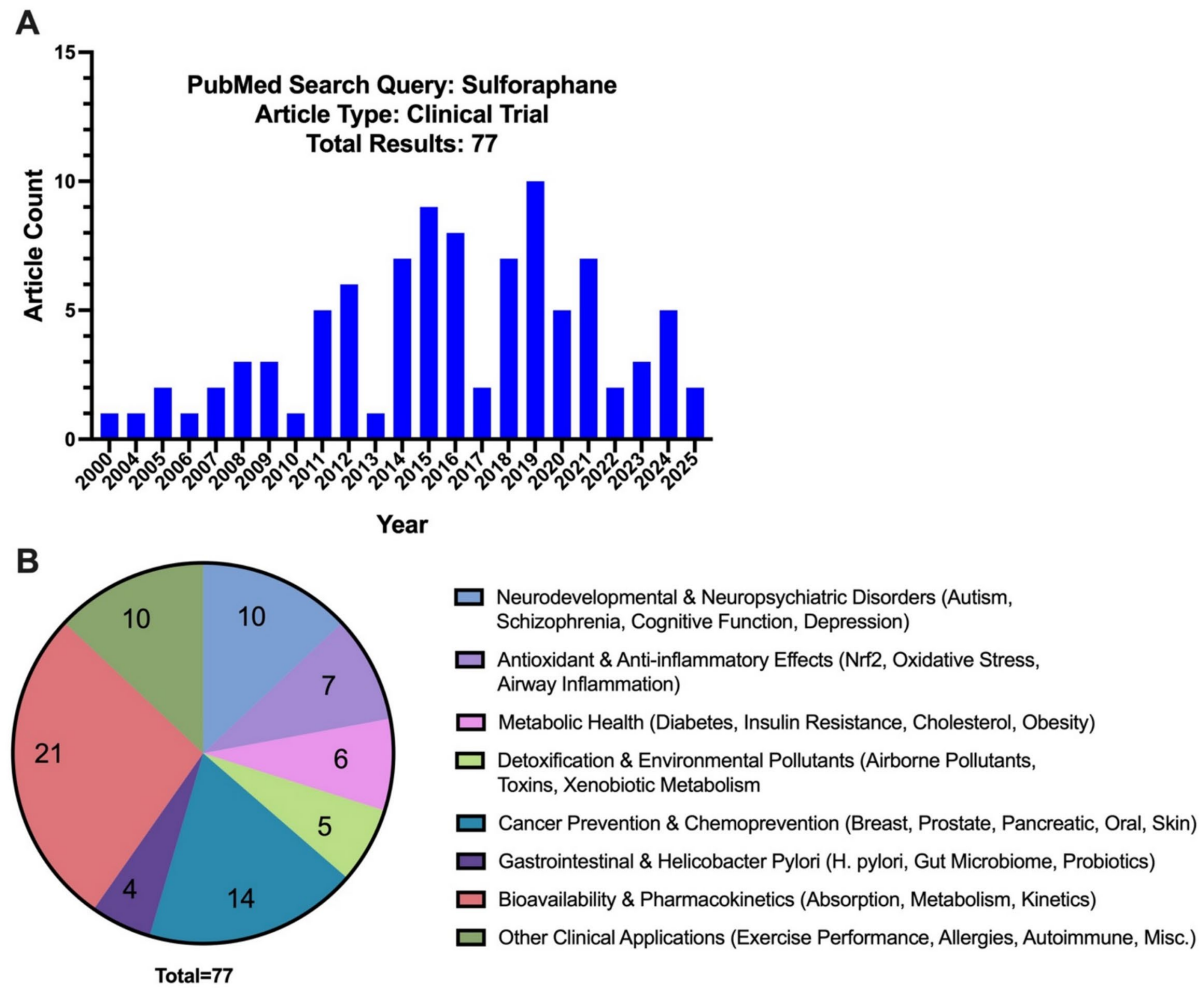
**(A)** PubMed search results for “sulforaphane” and “clinical trial” by the year and **(B)** by category.

The therapeutic potential of sulforaphane has gained increasing attention due to its ability to modulate oxidative stress and neuroinflammation. These properties studied in preclinical models suggest promising applications in neurodevelopmental and neurodegenerative disorders. Recent clinical trials investigated sulforaphane’s efficacy with a particular focus on autism spectrum disorders, schizophrenia, and neurodegenerative disorders. Currently there are 77 published reports from clinical trials on PubMed under the search results for sulforaphane, with the publication dates ranging from 2000 to 2025 (Figure 3 and Supplementary Table 1). Since 2020, 8 of the 20 published clinical trials have focused on neurodevelopmental and neuropsychiatric/cognitive disorders, exhibiting a significant rise in clinical trials exploring sulforaphane’s efficacy in the CNS compared to the previous two decades where only two of 57 trials focused on ASD alone with no published trials examining other CNS disorders (Supplementary Table 1).

Among the trials focusing on neurological outcomes, ASD has been the most extensively studied condition (6 clinical trials detailed in Table 1). Early clinical evidence (Singh et al., 2014) demonstrated that 18 weeks of sulforaphane-rich broccoli sprout extract treatment in young men with ASD significantly improved social responsiveness and behaviors assessed by the Aberrant Behavior Checklist (ABC) (−21.44 ± 4.34 total change in points from baseline sulforaphane; *p* < 0.0001) and Social Responsiveness Scale (SRS) (−20.40 ± 4.54; *p =* 0.017) (Singh et al., 2014). This study was pivotal in generating interest in sulforaphane as a potential intervention for ASD-related symptoms. However, subsequent trials yielded mixed findings. For example, a 12-week open-label study of Avmacol® tablets—containing sulforaphane precursor glucoraphanin and active myrosinase—(Bent et al., 2018) found improvements from baseline in both ABC [social withdrawal: − 3.0 (95% CI: − 5.6 to − 0.4); *p =* 0.02] and SRS scores [total: −9.7 (95% CI: − 18.7 to − 0.8); *p =* 0.03], communication [−5.0 (95% CI: − 8.4 to −1.5); *p =* 0.005] and motivation [−3.1 (95% CI: − 5.1 to − 1.2); *p =* 0.001] in patients with ages ranging from 7 to 21 years of age (Bent et al., 2018). However, a 36-week randomized controlled trial using BroccoPhane® broccoli sprout powder and red radish sprout powder (Magner et al., 2023) found no statistically significant changes in scores between sulforaphane and placebo groups in younger children ages 3–7 years of age (Magner et al., 2023). Similarly, a larger, multi-center trial using Avmacol® (Ou et al., 2024) showed mixed results, reporting no significant differences in caregiver ratings for ABC and SRS scores. However, they reported notable improvements in clinician-rated assessments, with patients ages 3–15 years of age showing significant improvement in the sulforaphane treatment group on the OSU Autism rating scale DSM-IV (OARS-4) in particular (Ou et al., 2024). Another study conducted by Momtazmanesh et al., 2020 showed clinical improvement in ASD patient irritability and hyperactivity outcomes when sulforaphane was used as an adjunct therapy to risperidone—an atypical antipsychotic—in patients aged 4–12 years of age (Momtazmanesh et al., 2020).

Two studies took a different approach from examining behavior alone and correlated behavioral outcomes with patient metabolic data. Bent et al. (2018) correlated behavioral improvements with urinary metabolites (Bent et al., 2018). They found negative correlations between clinical symptom scores and metabolites involved in redox metabolism, as well as neurotransmitters in patients treated with sulforaphane; meaning as the excretion of these metabolites increased, the clinical scores decreased indicating behavioral improvement (Bent et al., 2018). In another 15-week clinical trial (Zimmerman et al., 2021) in children from 3 to 12 years of age, researchers examined metabolites and biomarkers in patient plasma samples in addition to behavioral scores. Ultimately, they found no statistically significant improvements in total clinician-rated scores but saw trends towards improvement in patients taking Avmacol®. However, the caregiver ratings in this study for ABC and SRS improved significantly. Notably, this study showed evidence of sulforaphane’s biological effects, with significant reductions in IL-6 and TNFα in patients taking sulforaphane at week 15 (Zimmerman et al., 2021). Taken together, these trials highlight of both the potential of sulforaphane as a therapeutic agent for ASD and the limitations in the selected assays and clinical assessments. While several of the studies demonstrate behavioral improvement (Singh et al., 2014; Bent et al., 2018; Momtazmanesh et al., 2020), others report minimal or no significant effect (Magner et al., 2023; Ou et al., 2024; Zimmerman et al., 2021). Differences in study design, age of patient population, measured outcomes, and treatment dosage and duration likely contribute to inconsistent findings (Table 1). In addition to these limitations, there are significant differences in bioavailability depending on the preparation and delivery of sulforaphane, which is highest when glucoraphanin is administered as freeze-dried broccoli sprouts with active myrosinase either in capsule form or by pre-hydrolyzing it in juice (Fahey et al., 2015).

As for the remaining published clinical trials grouped in the neurodevelopmental and neuropsychiatric disorders category, only two targeted patients with schizophrenia (Dickerson et al., 2021; Huang et al., 2025), one examined depression, and the last focused on cognitive performance in older individuals. In patients with schizophrenia, overall symptoms were not resolved in the first study targeting the use of sulforaphane (Dickerson et al., 2021). However, high dose sulforaphane (1700 mg/day) in a following study significantly improved some of the negative symptoms of schizophrenia after 24 weeks (Huang et al., 2025). In the study examining depression, researchers saw improvements in the Hamilton Rating Scale for Depression—a 17-item rating scale for depression symptom severity—in patients taking sulforaphane following surgery for coronary artery bypass graft or percutaneous coronary intervention (Ghazizadeh-Hashemi et al., 2021). In healthy patients ages 60–80 years of age, sulforaphane supplementation improved processing speed and working memory compared to groups that received placebos, but did not see an additive effect with brain training and sulforaphane (Nouchi et al., 2021). Overall, these studies demonstrate the potential for sulforaphane to support cognitive and psychiatric health, although its effectiveness can be driven by dosage, patient population age, and concurrent interventions or treatments.

Future clinical trials on the efficacy of sulforaphane can improve our understanding of the patient populations that can benefit from the anti-oxidative and anti-inflammatory effects of sulforaphane. While there is currently a registered study to examine the effect of sulforaphane in patient populations with attention-deficit disorder with or without hyperactivity in children 6–12 years old (NCT06594536), as well as a study currently recruiting to evaluate the effects of sulforaphane in PD patients (NCT05084365), and a clinical study registered to examine AD (NCT04213391), there remains large gaps in the literature regarding the efficacy of sulforaphane as a treatment for neurological disorders.

## 6 Challenges and future directions

Despite promising evidence supporting the neuroprotective properties of sulforaphane, significant challenges must be addressed within its preclinical and clinical applications. For instance, many preclinical models administer sulforaphane simultaneously with an induced neuronal insult, creating an artificial scenario that may not reflect actual clinical applications. Treating a patient population already diagnosed with a neurodevelopmental or neurodegenerative disorder presents greater challenges than what is presented in a laboratory, since these patients will present with varying levels of disease severity and/or progression with associated cellular damage and neuroinflammation. However, sulforaphane’s origin in cancer research highlights one of its greatest strength—prevention. As a preventative agent, studies indicate beneficial effects of low doses of sulforaphane (Table 2). Sulforaphane and glucoraphanin were first identified in cruciferous vegetables after epidemiologic studies linked a diet rich in these foods with a lowered risk of colon cancer. As we have highlighted in this review, extensive research has since demonstrated the ability of sulforaphane-mediated NRF2 activation to prevent oxidative stress and neuroinflammation—key contributors to both neurodevelopmental and neurodegenerative disorders. Thus, while sulforaphane has shown some potential to lessen behaviors associated with established disease pathologies, its strongest effects in preclinical models appear to be involved in reducing disease risk before onset of pathological manifestations.

Thus, there are several considerations when contemplating clinical applications of sulforaphane. Specifically, neurological disorders often present with chronic inflammation and oxidative stress that may benefit from higher sulforaphane doses. Thus, the severity of the disorder may impact sulforaphane’s effectiveness at treating neurological conditions. Furthermore, differences in the bioavailability of different sulforaphane preparations can limit efficacy. These considerations likely contribute to discrepancies in sulforaphane preclinical trials (Table 1). Finally, pharmacokinetic properties of other synthetic and natural NRF2 activators may be more suitable for specific neurological conditions (Robledinos-Antón et al., 2019). However, we focused this review on sulforaphane due to its overwhelming use in neuronal disease models and clinical trials. Finally, while sulforaphane is generally well-tolerated at low dose, clinical trials as higher sulforaphane doses can have unwanted side effects, such as lethargy, hypothermia, and gastrointestinal distress, although these generally occur at very high doses (Yagishita et al., 2019). These side effects will have to be taken into consideration when determining appropriate dosing for chronic disorders.

It is quite thought-provoking that sulforaphane, a compound originally recognized for its chemopreventative properties, is now increasingly regarded for its neuroprotective potential in a wide array of CNS disorders. Initially identified for its ability to induce phase II detoxification enzymes and inhibit carcinogenesis, sulforaphane exerted its neuroprotective effects by triggering NRF2-dependent adaptive stress responses—a classic example of hormesis (Butterfield et al., 2023; Bondy, 2023; Calabrese and Kozumbo, 2021). Hormesis refers to a biphasic, dose-dependent phenomenon where low doses elicit beneficial cellular response, while high doses become detrimental to cell function or are toxic. What is particularly interesting about sulforaphane is the differential sensitivity of distinct cell types to this hormetic effect (Calabrese and Kozumbo, 2021). In cancer biology, malignant cells exhibit altered redox balance and enhanced antioxidants, rendering them resistant to ROS during cancer progression (Xing et al., 2022). Consequently, higher concentrations of sulforaphane are necessary to induce cell cycle arrest and apoptosis, which are desirable outcomes in a chemotherapeutic context of inhibition. Alternatively, neuronal cells are seemingly more susceptible to sulforaphane’s activity. At lower, sub-toxic concentrations, sulforaphane increased NRF2-mediated antioxidant defenses, ultimately enhancing neuronal survival in cell culture models driven by oxidative and inflammatory insults (Table 2) (Vauzour et al., 2010; Sandouka and Shekh-Ahmad, 2021; Hou et al., 2018; Wu et al., 2012; Bertuccio et al., 2024). However, in many cases these neuronal cells are vulnerable to sulforaphane-induced toxicity at concentrations well-tolerated by cancer cells (Vauzour et al., 2010; Wu et al., 2012; Calabrese and Kozumbo, 2021; Bertuccio et al., 2024). Therefore, sulforaphane-based research and implementation of therapeutic strategies must be carefully tailored to the unique biological sensitivities of target tissues.

## References

[ref1] Abiko Y. Miura T. Phuc B. H. Shinkai Y. Kumagai Y. (2011). Participation of covalent modification of Keap1 in the activation of Nrf2 by tert-butylbenzoquinone, an electrophilic metabolite of butylated hydroxyanisole. Toxicol. Appl. Pharmacol. 255, 32–39. doi: 10.1016/j.taap.2011.05.013, PMID: 21651925

[ref2] Aguiar C. C. T. Almeida A. B. Araújo P. V. P. Chaves E. M. C. Vale O. C.. (2012). Oxidative stress and epilepsy. Literature Rev. Oxid Med Cell Longev. 2012:795259. doi: 10.1155/2012/795259 PMC3403512 22848783

[ref3] Anilkumar S. Wright-Jin E. (2024). NF-κB as an inducible regulator of inflammation in the central nervous system. Cells 13:485. doi: 10.3390/cells13060485, PMID: 38534329 PMC10968931

[ref4] Arriagada P. V. Growdon J. H. Hedley-Whyte E. T. Hyman B. T. (1992). Neurofibrillary tangles but not senile plaques parallel duration and severity of Alzheimer’s disease. Neurology 42:631. doi: 10.1212/WNL.42.3.631, PMID: 1549228

[ref5] Arruebarrena Di Palma A. Perk E. A. Carboni M. E. García-Mata C. Budak H. Tör M.. (2022). The isothiocyanate sulforaphane induces respiratory burst oxidase homologue D-dependent reactive oxygen species production and regulates expression of stress response genes. Plant. Direct 6:e437. doi: 10.1002/pld3.437, PMID: 36091879 PMC9448665

[ref6] Averill-Bates D. (2024). Reactive oxygen species and cell signaling. Biochim. Biophys. Acta BBA - Mol. Cell Res. 1871:119573. doi: 10.1016/j.bbamcr.2023.119573 37949302

[ref7] Bent S. Lawton B. Warren T. Widjaja F. Dang K. Fahey J. W.. (2018). Identification of urinary metabolites that correlate with clinical improvements in children with autism treated with sulforaphane from broccoli. Mol. Autism. 9:35. doi: 10.1186/s13229-018-0218-4, PMID: 29854372 PMC5975568

[ref8] Bertuccio M. P. Saija C. Acri G. Ientile R. Caccamo D. Currò M. (2024). Sulforaphane effects on neuronal-like cells and peripheral blood mononuclear cells exposed to 2.45 GHz electromagnetic radiation. Int. J. Mol. Sci. 25:7872. doi: 10.3390/ijms25147872, PMID: 39063113 PMC11276899

[ref9] Bessetti R. N. Cobb M. Lilley R. M. Johnson N. Z. Perez D. A. Koonce V. M.. (2025). Sulforaphane protects developing neural networks from VPA-induced synaptic alterations. Mol. Psychiatry 2, 1–17. doi: 10.1038/s41380-025-02967-5 PMC12339368 40175519

[ref10] Bjørklund G. Meguid N. A. El-Bana M. A. Tinkov A. A. Saad K. Dadar M.. (2020). Oxidative stress in autism Spectrum disorder. Mol. Neurobiol. 57, 2314–2332. doi: 10.1007/s12035-019-01742-2, PMID: 32026227

[ref11] Blevins H. M. Xu Y. Biby S. Zhang S. (2022). The NLRP3 inflammasome pathway: a review of mechanisms and inhibitors for the treatment of inflammatory diseases. Front. Aging Neurosci. 14:879021. doi: 10.3389/fnagi.2022.879021 35754962 PMC9226403

[ref12] Bloom G. S. (2014). Amyloid-β and tau: the trigger and bullet in Alzheimer disease pathogenesis. JAMA Neurol. 71, 505–508. doi: 10.1001/jamaneurol.2013.5847, PMID: 24493463 PMC12908160

[ref13] Bobermin L. D. Weber F. B. dos Santos T. M. Belló-Klein A. Wyse A. T. S. Gonçalves C. A.. (2020). Sulforaphane induces glioprotection after LPS challenge. Cell. Mol. Neurobiol. 42, 829–846. doi: 10.1007/s10571-020-00981-5, PMID: 33079284 PMC11441213

[ref14] Bondy S. C. (2023). The hormesis concept: strengths and shortcomings. Biomol. Ther. 13:1512. doi: 10.3390/biom13101512, PMID: 37892194 PMC10604602

[ref15] Bošković M. Vovk T. Kores Plesničar B. Grabnar I. (2011). Oxidative stress in schizophrenia. Curr. Neuropharmacol. 9, 301–312. doi: 10.2174/157015911795596595, PMID: 22131939 PMC3131721

[ref16] Brembati V. Faustini G. Longhena F. Bellucci A. (2023). Alpha synuclein post translational modifications: potential targets for Parkinson’s disease therapy? Front. Mol. Neurosci. 16:1197853. doi: 10.3389/fnmol.2023.1197853 37305556 PMC10248004

[ref17] Brown A. S. Sourander A. Hinkka-Yli-Salomäki S. McKeague I. W. Sundvall J. Surcel H. M. (2014). Elevated maternal C-reactive protein and autism in a national birth cohort. Mol. Psychiatry 19, 259–264. doi: 10.1038/mp.2012.197, PMID: 23337946 PMC3633612

[ref18] Buczyńska A. Sidorkiewicz I. Krętowski A. J. Zbucka-Krętowska M. (2023). The role of oxidative stress in trisomy 21 phenotype. Cell. Mol. Neurobiol. 43, 3943–3963. doi: 10.1007/s10571-023-01417-6, PMID: 37819608 PMC10661812

[ref19] Butterfield D. A. Boyd-Kimball D. Reed T. T. (2023). Cellular stress response (hormesis) in response to bioactive nutraceuticals with relevance to Alzheimer disease. Antioxid. Redox Signal. 38, 643–669. doi: 10.1089/ars.2022.0214, PMID: 36656673 PMC10025851

[ref9001] Cai Y. X. Augustin M. A. Jegasothy H. Wang J. H. Terefe N. S. (2020). Mild heat combined with lactic acid fermentation: a novel approach for enhancing sulforaphane yield in broccoli puree. Food Funct. 11, 779–786. doi: 10.1039/C9FO02089F 31922158

[ref20] Calabrese E. J. Kozumbo W. J. (2021). The phytoprotective agent sulforaphane prevents inflammatory degenerative diseases and age-related pathologies via Nrf2-mediated hormesis. Pharmacol. Res. 163:105283. doi: 10.1016/j.phrs.2020.105283, PMID: 33160067

[ref21] Carrasco-Pozo C. Tan K. N. Borges K. (2015). Sulforaphane is anticonvulsant and improves mitochondrial function. J. Neurochem. 135, 932–942. doi: 10.1111/jnc.13361, PMID: 26365487

[ref22] Chang Y. T. Chang W. N. Tsai N. W. Huang C. C. Kung C. T. Su Y. J.. (2014). The roles of biomarkers of oxidative stress and antioxidant in Alzheimer’s disease: a systematic review. Biomed. Res. Int. 2014:182303, 1–14. doi: 10.1155/2014/182303 24949424 PMC4053273

[ref23] Cheignon C. Tomas M. Bonnefont-Rousselot D. Faller P. Hureau C. Collin F. (2017). Oxidative stress and the amyloid beta peptide in Alzheimer’s disease. Redox Biol. 14, 450–464. doi: 10.1016/j.redox.2017.10.014 29080524 PMC5680523

[ref24] Cobley J. N. Fiorello M. L. Bailey D. M. (2018). 13 reasons why the brain is susceptible to oxidative stress. Redox Biol. 15, 490–503. doi: 10.1016/j.redox.2018.01.008, PMID: 29413961 PMC5881419

[ref25] Colditz G. Branch L. Lipnick R. Willett W. Rosner B. Posner B.. (1985). Increased green and yellow vegetable intake and lowered cancer deaths in an elderly population. Am. J. Clin. Nutr. 41, 32–36. doi: 10.1093/ajcn/41.1.32, PMID: 3966422

[ref26] Cui H. Elford J. D. Alitalo O. Perez-Pardo P. Tampio J. Huttunen K. M.. (2023). Nigrostriatal 6-hydroxydopamine lesions increase alpha-synuclein levels and permeability in rat colon. Neurobiol. Aging 129, 62–71. doi: 10.1016/j.neurobiolaging.2023.05.007, PMID: 37271045

[ref27] D’Mello A. M. Stoodley C. J. (2015). Cerebro-cerebellar circuits in autism spectrum disorder. Front. Neurosci. 9:408. doi: 10.3389/fnins.2015.00408 26594140 PMC4633503

[ref28] Daněk J. Danačíková Š. Kala D. Svoboda J. Kapoor S. Pošusta A.. (2022). Sulforaphane ameliorates metabolic changes associated with status epilepticus in immature rats. Front. Cell. Neurosci. 16:855161. doi: 10.3389/fncel.2022.855161 35370554 PMC8965559

[ref29] Darwish S. F. Elbadry A. M. M. Elbokhomy A. S. Salama G. A. Salama R. M. (2023). The dual face of microglia (M1/M2) as a potential target in the protective effect of nutraceuticals against neurodegenerative diseases. Front. Aging 4. doi: 10.3389/fragi.2023.1231706 PMC10513083 37744008

[ref30] De Felice C. Signorini C. Leoncini S. Pecorelli A. Durand T. Valacchi G.. (2012). The role of oxidative stress in Rett syndrome: an overview. Ann. N. Y. Acad. Sci. 1259, 121–135. doi: 10.1111/j.1749-6632.2012.06611.x, PMID: 22758644

[ref31] Della Bianca V. Dusi S. Bianchini E. Dal Prà I. Rossi F. (1999). Β-Amyloid activates the O⨪2 forming NADPH oxidase in microglia, monocytes, and neutrophils. J. Biol. Chem. 274, 15493–15499. doi: 10.1074/jbc.274.22.15493, PMID: 10336441

[ref32] Dhakshinamoorthy S. Jaiswal A. K. (2001). Functional characterization and role of INrf2 in antioxidant response element-mediated expression and antioxidant induction of NAD(P)H:quinone oxidoreductase1 gene. Oncogene 20, 3906–3917. doi: 10.1038/sj.onc.1204506, PMID: 11439354

[ref33] Dias V. Junn E. Mouradian M. M. (2013). The role of oxidative stress in Parkinson’s disease. J. Parkinsons Dis. 3, 461–491. doi: 10.3233/JPD-130230, PMID: 24252804 PMC4135313

[ref34] Dickerson F. Origoni A. Katsafanas E. Squire A. Newman T. Fahey J.. (2021). Randomized controlled trial of an adjunctive sulforaphane nutraceutical in schizophrenia. Schizophr. Res. 231, 142–144. doi: 10.1016/j.schres.2021.03.018, PMID: 33839372

[ref35] DiSabato D. Quan N. Godbout J. P. (2016). Neuroinflammation: the devil is in the details. J. Neurochem. 139, 136–153. doi: 10.1111/jnc.13607 26990767 PMC5025335

[ref36] Endo N. Makinodan M. Somayama N. Komori T. Kishimoto T. Nishi M. (2019). Characterization of behavioral phenotypes in the BTBR T+ *Itpr3tf*/J mouse model of autism spectrum disorder under social housing conditions using the multiple animal positioning system. Exp. Anim. 68, 319–330. doi: 10.1538/expanim.18-0177, PMID: 30905912 PMC6699967

[ref37] Eren E. Tufekci K. U. Isci K. B. Tastan B. Genc K. Genc S. (2018). Sulforaphane inhibits lipopolysaccharide-induced inflammation, cytotoxicity, oxidative stress, and miR-155 expression and switches to Mox phenotype through activating extracellular signal-regulated kinase 1/2–nuclear factor erythroid 2-related factor 2/antioxidant response element pathway in murine microglial cells. Front. Immunol. 9:36. doi: 10.3389/fimmu.2018.00036 29410668 PMC5787131

[ref38] Eskandani M. Hamishehkar H. Ezzati Nazhad Dolatabadi J. (2014). Cytotoxicity and DNA damage properties of tert-butylhydroquinone (TBHQ) food additive. Food Chem. 153, 315–320. doi: 10.1016/j.foodchem.2013.12.087, PMID: 24491735

[ref39] Fahey J. W. Holtzclaw W. D. Wehage S. L. Wade K. L. Stephenson K. K. Talalay P. (2015). Sulforaphane bioavailability from glucoraphanin-rich broccoli: control by active endogenous Myrosinase. PLoS One 10:e0140963. doi: 10.1371/journal.pone.0140963, PMID: 26524341 PMC4629881

[ref40] Fahey J. W. Zhang Y. Talalay P. (1997). Broccoli sprouts: An exceptionally rich source of inducers of enzymes that protect against chemical carcinogens. Proc. Natl. Acad. Sci. 94, 10367–10372. doi: 10.1073/pnas.94.19.10367, PMID: 9294217 PMC23369

[ref41] Fei Y. Shi R. Song Z. Wu J. (2020). Metabolic control of epilepsy: a promising therapeutic target for epilepsy. Front. Neurol. 11:592514. doi: 10.3389/fneur.2020.592514, PMID: 33363507 PMC7753014

[ref42] Fenwick G. R. Heaney R. K. Mullin W. J. VanEtten C. H. (1983). Glucosinolates and their breakdown products in food and food plants. Crit. Rev. Food Sci. Nutr. 18, 123–201. doi: 10.1080/10408398209527361, PMID: 6337782

[ref43] Ferber E. Gerhards J. Sauer M. Krischke M. Dittrich M. T. Müller T.. (2020). Chemical priming by isothiocyanates protects against intoxication by products of the mustard oil bomb. Front. Plant Sci. 11:887. doi: 10.3389/fpls.2020.00887 32676087 PMC7333730

[ref44] Folbergrová J. Ješina P. Otáhal J. (2023). Protective effect of sulforaphane on oxidative stress and mitochondrial dysfunction associated with status epilepticus in immature rats. Mol. Neurobiol. 60, 2024–2035. doi: 10.1007/s12035-022-03201-x, PMID: 36598650 PMC9984354

[ref45] Fujita Y. Fujita A. Ishima T. Hirai A. Suzuki S. Suganuma H.. (2020). Dietary intake of glucoraphanin during pregnancy and lactation prevents the behavioral abnormalities in the offspring after maternal immune activation. Neuropsychopharmacol Rep. 40, 268–274. doi: 10.1002/npr2.12112, PMID: 32463181 PMC7722647

[ref46] Fuse Y. Kobayashi M. (2017). Conservation of the Keap1-Nrf2 system: an evolutionary journey through stressful space and time. Molecules 22:436. doi: 10.3390/molecules22030436, PMID: 28282941 PMC6155405

[ref47] Michael G. George K. (2022). A case of hypomagnesemia presenting as new-onset seizure. Cureus 14, 1–14. doi: 10.7759/cureus.23791 PMC9064401 35518545

[ref48] Gacesa R. Dunlap W. C. Barlow D. J. Laskowski R. A. Long P. F. (2016). Rising levels of atmospheric oxygen and evolution of Nrf2. Sci. Rep. 6:27740. doi: 10.1038/srep27740, PMID: 27297177 PMC4906274

[ref49] Galuppo M. Iori R. De Nicola G. R. Bramanti P. Mazzon E. (2013). Anti-inflammatory and anti-apoptotic effects of (*RS*)-glucoraphanin bioactivated with myrosinase in murine sub-acute and acute MPTP-induced Parkinson’s disease. Bioorg. Med. Chem. 21, 5532–5547. doi: 10.1016/j.bmc.2013.05.065, PMID: 23810671

[ref50] Gao C. Jiang J. Tan Y. Chen S. (2023). Microglia in neurodegenerative diseases: mechanism and potential therapeutic targets. Signal Transduct. Target. Ther. 8, 1–37. doi: 10.1038/s41392-023-01588-0 37735487 PMC10514343

[ref51] Gella A. Durany N. (2009). Oxidative stress in Alzheimer disease. Cell Adhes. Migr. 3, 88–93. doi: 10.4161/cam.3.1.7402, PMID: 19372765 PMC2675154

[ref52] Gharavi N. Haggarty S. El-Kadi A. O. S. (2007). Chemoprotective and carcinogenic effects of tert-butylhydroquinone and its metabolites. Curr. Drug Metab. 8, 1–7. 17266519 10.2174/138920007779315035

[ref53] Ghazizadeh-Hashemi F. Bagheri S. Ashraf-Ganjouei A. Moradi K. Shahmansouri N. Mehrpooya M.. (2021). Efficacy and safety of sulforaphane for treatment of mild to moderate depression in patients with history of cardiac interventions: a randomized, double-blind, placebo-controlled clinical trial. Psychiatry Clin. Neurosci. 75, 250–255. doi: 10.1111/pcn.13276, PMID: 34033171

[ref54] Ginhoux F. Prinz M. (2015). Origin of microglia: current concepts and past controversies. Cold Spring Harb. Perspect. Biol. 7:a020537. doi: 10.1101/cshperspect.a020537, PMID: 26134003 PMC4526747

[ref55] Goines P. E. Croen L. A. Braunschweig D. Yoshida C. K. Grether J. Hansen R.. (2011). Increased midgestational IFN-γ, IL-4 and IL-5 in women bearing a child with autism: a case-control study. Mol. Autism. 2:13. doi: 10.1186/2040-2392-2-13, PMID: 21810230 PMC3170586

[ref56] Graham S. Dayal H. Swanson M. Mittelman A. Wilkinson G. (1978). Diet in the epidemiology of cancer of the colon and rectum2. JNCI J. Natl. Cancer Inst. 61, 709–714. 278848

[ref57] Grundke-Iqbal I. Iqbal K. Tung Y. C. Quinlan M. Wisniewski H. M. Binder L. I. (1986). Abnormal phosphorylation of the microtubule-associated protein tau (tau) in Alzheimer cytoskeletal pathology. Proc. Natl. Acad. Sci. USA 83, 4913–4917. doi: 10.1073/pnas.83.13.4913, PMID: 3088567 PMC323854

[ref58] Guo S. Wang H. Yin Y. (2022). Microglia polarization from M1 to M2 in neurodegenerative diseases. Front. Aging Neurosci. 14:815347. doi: 10.3389/fnagi.2022.815347 35250543 PMC8888930

[ref59] Habas A. Hahn J. Wang X. Margeta M. (2013). Neuronal activity regulates astrocytic Nrf2 signaling. Proc. Natl. Acad. Sci. USA 110, 18291–18296. doi: 10.1073/pnas.1208764110, PMID: 24145448 PMC3831500

[ref60] Han V. X. Patel S. Jones H. F. Dale R. C. (2021). Maternal immune activation and neuroinflammation in human neurodevelopmental disorders. Nat. Rev. Neurol. 17, 564–579. doi: 10.1038/s41582-021-00530-8, PMID: 34341569

[ref61] Heiss E. Herhaus C. Klimo K. Bartsch H. Gerhäuser C. (2001). Nuclear factor κB is a molecular target for sulforaphane-mediated anti-inflammatory mechanisms *. J. Biol. Chem. 276, 32008–32015. doi: 10.1074/jbc.M104794200, PMID: 11410599

[ref62] Hou T. T. Yang H. Y. Wang W. Wu Q. Q. Tian Y. R. Jia J. P. (2018). Sulforaphane inhibits the generation of amyloid-β oligomer and promotes spatial learning and memory in Alzheimer’s disease (PS1V97L) transgenic mice. J. Alzheimers Dis. 62, 1803–1813. doi: 10.3233/JAD-171110, PMID: 29614663

[ref63] Huang J. Chen A. Jin H. Liu F. Hei G. Teng Z.. (2025). Efficacy and safety of sulforaphane added to antipsychotics for the treatment of negative symptoms of schizophrenia: a randomized controlled trial. J. Clin. Psychiatry 86:24m15272. doi: 10.4088/JCP.24m15272 39832347

[ref64] Huber C. M. Yee C. May T. Dhanala A. Mitchell C. S. (2018). Cognitive decline in preclinical Alzheimer’s disease: amyloid-Beta versus tauopathy. J. Alzheimers Dis. 61, 265–281. doi: 10.3233/JAD-170490, PMID: 29154274 PMC5734131

[ref65] Imhoff B. R. Hansen J. M. (2010). Tert-butylhydroquinone induces mitochondrial oxidative stress causing Nrf2 activation. Cell Biol. Toxicol. 26, 541–551. doi: 10.1007/s10565-010-9162-6, PMID: 20429028

[ref66] Innamorato N. G. Rojo A. I. García-Yagüe Á. J. Yamamoto M. de Ceballos M. L. Cuadrado A. (2008). The transcription factor Nrf2 is a therapeutic target against brain inflammation1. J. Immunol. 181, 680–689. doi: 10.4049/jimmunol.181.1.680 18566435

[ref67] Itoh K. Chiba T. Takahashi S. Ishii T. Igarashi K. Katoh Y.. (1997). An Nrf2/small Maf heterodimer mediates the induction of phase II detoxifying enzyme genes through antioxidant response elements. Biochem. Biophys. Res. Commun. 236, 313–322. doi: 10.1006/bbrc.1997.6943, PMID: 9240432

[ref68] Itoh K. Wakabayashi N. Katoh Y. Ishii T. Igarashi K. Engel J. D.. (1999). Keap1 represses nuclear activation of antioxidant responsive elements by Nrf2 through binding to the amino-terminal Neh2 domain. Genes Dev. 13, 76–86. doi: 10.1101/gad.13.1.76, PMID: 9887101 PMC316370

[ref69] Jazwa A. Rojo A. I. Innamorato N. G. Hesse M. Fernández-Ruiz J. Cuadrado A. (2011). Pharmacological targeting of the transcription factor Nrf2 at the basal ganglia provides disease modifying therapy for experimental parkinsonism. Antioxid. Redox Signal. 14, 2347–2360. doi: 10.1089/ars.2010.3731, PMID: 21254817

[ref70] Jiang S. Maphis N. M. Binder J. Chisholm D. Weston L. Duran W.. (2021). Proteopathic tau primes and activates interleukin-1β via myeloid-cell-specific MyD88-and NLRP3-ASC-inflammasome pathway. Cell Rep. 36:109720. doi: 10.1016/j.celrep.2021.109720, PMID: 34551296 PMC8491766

[ref71] Jones K. L. Croen L. A. Yoshida C. K. Heuer L. Hansen R. Zerbo O.. (2017). Autism with intellectual disability is associated with increased levels of maternal cytokines and chemokines during gestation. Mol. Psychiatry 22, 273–279. doi: 10.1038/mp.2016.77, PMID: 27217154 PMC5122473

[ref72] Juan C. A. Pérez de la Lastra J. M. Plou F. J. Pérez-Lebeña E. (2021). The chemistry of reactive oxygen species (ROS) revisited: outlining their role in biological macromolecules (DNA, lipids and proteins) and induced pathologies. Int. J. Mol. Sci. 22:4642. doi: 10.3390/ijms22094642 33924958 PMC8125527

[ref73] Jurga A. M. Paleczna M. Kuter K. Z. (2020). Overview of general and discriminating markers of differential microglia phenotypes. Front. Cell. Neurosci. 14:198. doi: 10.3389/fncel.2020.00198, PMID: 32848611 PMC7424058

[ref74] Kraft A. D. Harry G. J. (2011). Features of microglia and neuroinflammation relevant to environmental exposure and neurotoxicity. Int. J. Environ. Res. Public Health 8, 2980–3018. doi: 10.3390/ijerph8072980, PMID: 21845170 PMC3155341

[ref75] Kraft A. D. Johnson D. A. Johnson J. A. (2004). Nuclear factor E2-related factor 2-dependent antioxidant response element activation by tert-butylhydroquinone and sulforaphane occurring preferentially in astrocytes conditions neurons against oxidative insult. J. Neurosci. 24, 1101–1112. doi: 10.1523/JNEUROSCI.3817-03.2004, PMID: 14762128 PMC6793572

[ref76] Kumar V. (2019). Toll-like receptors in the pathogenesis of neuroinflammation. J. Neuroimmunol. 332, 16–30. doi: 10.1016/j.jneuroim.2019.03.012, PMID: 30928868

[ref77] Kurutas E. B. (2016). The importance of antioxidants which play the role in cellular response against oxidative/nitrosative stress: current state. Nutr. J. 15:71. doi: 10.1186/s12937-016-0186-5 27456681 PMC4960740

[ref78] Lee C. (2017). Collaborative power of Nrf2 and PPARγ activators against metabolic and drug-induced oxidative injury. Oxidative Med. Cell. Longev. 2017:1378175. doi: 10.1155/2017/1378175, PMID: 28928902 PMC5591982

[ref79] Lee S. Choi B. R. Kim J. LaFerla F. M. Park J. H. Y. Han J. S.. (2018). Sulforaphane upregulates the heat shock protein co-chaperone CHIP and clears amyloid-β and tau in a mouse model of Alzheimer’s disease. Mol. Nutr. Food Res. 62:1800240. doi: 10.1002/mnfr.201800240, PMID: 29714053

[ref80] Lee D. Lal N. K. Lin Z. J. D. Ma S. Liu J. Castro B.. (2020). Regulation of reactive oxygen species during plant immunity through phosphorylation and ubiquitination of RBOHD. Nat. Commun. 11:1838. doi: 10.1038/s41467-020-15601-5, PMID: 32296066 PMC7160206

[ref81] Li Q. Fadoul G. Ikonomovic M. Yang T. Zhang F. (2022). Sulforaphane promotes white matter plasticity and improves long-term neurological outcomes after ischemic stroke via the Nrf2 pathway. Free Radic. Biol. Med. 193, 292–303. doi: 10.1016/j.freeradbiomed.2022.10.001, PMID: 36244590 PMC12335009

[ref82] Li W. Kong A. N. (2009). Molecular mechanisms of Nrf2-mediated antioxidant response. Mol. Carcinog. 48, 91–104. doi: 10.1002/mc.20465, PMID: 18618599 PMC2631094

[ref83] Li D. Pan Q. Xiao Y. Hu K. (2024). Advances in the study of phencyclidine-induced schizophrenia-like animal models and the underlying neural mechanisms. Schizophrenia. 10, 1–10. doi: 10.1038/s41537-024-00485-x, PMID: 39039065 PMC11263595

[ref84] Li C. Zhang L. Li X. Hu Q. Mao L. Shao Y.. (2025). Sulforaphane suppresses aβ accumulation and tau hyperphosphorylation in vascular cognitive impairment(VCI). J. Nutr. Biochem. 136:109803. doi: 10.1016/j.jnutbio.2024.109803, PMID: 39551165

[ref85] Lin M. Liu N. Qin Z. hong Wang Y. (2022). Mitochondrial-derived damage-associated molecular patterns amplify neuroinflammation in neurodegenerative diseases. Acta Pharmacol. Sin. 43, 2439–2447. doi: 10.1038/s41401-022-00879-6, PMID: 35233090 PMC9525705

[ref86] Litwa K. (2022). Shared mechanisms of neural circuit disruption in tuberous sclerosis across lifespan: bridging neurodevelopmental and neurodegenerative pathology. Front. Genet. 13:997461. doi: 10.3389/fgene.2022.997461 36506334 PMC9732432

[ref87] Liu J. Chandaka G. K. Zhang R. Parfenova H. (2020). Acute antioxidant and cytoprotective effects of sulforaphane in brain endothelial cells and astrocytes during inflammation and excitotoxicity. Pharmacol. Res. Perspect. 8:e00630. doi: 10.1002/prp2.630, PMID: 32715644 PMC7383090

[ref88] Liu X. Lin J. Zhang H. Khan N. U. Zhang J. Tang X.. (2022). Oxidative stress in autism spectrum disorder—current progress of mechanisms and biomarkers. Front. Psych. 13:813304. doi: 10.3389/fpsyt.2022.813304 PMC8921264 35299821

[ref89] Lobo-Silva D. Carriche G. M. Castro A. G. Roque S. Saraiva M. (2016). Balancing the immune response in the brain: IL-10 and its regulation. J. Neuroinflammation 13:297. doi: 10.1186/s12974-016-0763-8, PMID: 27881137 PMC5121946

[ref90] Ma Q. Kinneer K. Bi Y. Chan J. Y. Kan Y. W. (2004). Induction of murine NAD(P)H:quinone oxidoreductase by 2,3,7,8-tetrachlorodibenzo-p-dioxin requires the CNC (cap n collar) basic leucine zipper transcription factor Nrf2 (nuclear factor erythroid 2-related factor 2): cross-interaction between AhR (aryl hydrocarbon receptor) and Nrf2 signal transduction. Biochem. J. 377, 205–213. doi: 10.1042/BJ20031123, PMID: 14510636 PMC1223846

[ref91] Magner M. Thorová K. Župová V. Houška M. Švandová I. Novotná P.. (2023). Sulforaphane treatment in children with autism: a prospective randomized double-blind study. Nutrients 15:718. doi: 10.3390/nu15030718, PMID: 36771424 PMC9920098

[ref9002] Matusheski N. V. Juvik J. A. Jeffery E. H. (2004). Heating decreases epithiospecifier protein activity and increases sulforaphane formation in broccoli. Phytochemistry 65, 1273–1281. doi: 10.1016/j.phytochem.2004.04.013 15184012

[ref92] Medeiros R. Baglietto-Vargas D. LaFerla F. M. (2010). The role of tau in Alzheimer’s disease and related disorders. CNS Neurosci. Ther. 17, 514–524. doi: 10.1111/j.1755-5949.2010.00177.x 20553310 PMC4072215

[ref93] Meredith G. E. Rademacher D. J. (2011). MPTP mouse models of Parkinson’s disease: an update. J. Parkinsons Dis. 1, 19–33. doi: 10.3233/JPD-2011-11023, PMID: 23275799 PMC3530193

[ref94] Minogue A. M. (2017). Role of infiltrating monocytes/macrophages in acute and chronic neuroinflammation: effects on cognition, learning and affective behaviour. Prog. Neuro-Psychopharmacol. Biol. Psychiatry 79, 15–18. doi: 10.1016/j.pnpbp.2017.02.008, PMID: 28189704

[ref95] Momtazmanesh S. Amirimoghaddam-Yazdi Z. Moghaddam H. S. Mohammadi M. R. Akhondzadeh S. (2020). Sulforaphane as an adjunctive treatment for irritability in children with autism spectrum disorder: a randomized, double-blind, placebo-controlled clinical trial. Psychiatry Clin. Neurosci. 74, 398–405. doi: 10.1111/pcn.13016, PMID: 32347624

[ref96] Morroni F. Tarozzi A. Sita G. Bolondi C. Zolezzi Moraga J. M. Cantelli-Forti G.. (2013). Neuroprotective effect of sulforaphane in 6-hydroxydopamine-lesioned mouse model of Parkinson’s disease. Neurotoxicology 36, 63–71. doi: 10.1016/j.neuro.2013.03.004, PMID: 23518299

[ref97] Müller N. Weidinger E. Leitner B. Schwarz M. J. (2015). The role of inflammation in schizophrenia. Front. Neurosci. 9:372. doi: 10.3389/fnins.2015.00372, PMID: 26539073 PMC4612505

[ref98] Murphy M. P. LeVine H. (2010). Alzheimer’s disease and the β-amyloid peptide. J. Alzheimers Dis. JAD 19:311. doi: 10.3233/JAD-2010-122 20061647 PMC2813509

[ref99] Nadeem A. Ahmad S. F. Al-Harbi N. O. Attia S. M. Bakheet S. A. Ibrahim K. E.. (2019). Nrf2 activator, sulforaphane ameliorates autism-like symptoms through suppression of Th17 related signaling and rectification of oxidant-antioxidant imbalance in periphery and brain of BTBR T+tf/J mice. Behav. Brain Res. 364, 213–224. doi: 10.1016/j.bbr.2019.02.031, PMID: 30790585

[ref100] Nadler V. Kainic J. (1981). Acid as a tool for the study of temporal lobe epilepsy. Life Sci. 29, 2031–2042. 7031398 10.1016/0024-3205(81)90659-7

[ref101] Nouchi R. Hu Q. Saito T. Kawata NY dos S. Nouchi H. Kawashima R. (2021). Brain training and sulforaphane intake interventions separately improve cognitive performance in healthy older adults, whereas a combination of these interventions does not have more beneficial effects: evidence from a randomized controlled trial. Nutrients 13:352. doi: 10.3390/nu13020352 33503851 PMC7912304

[ref102] Oeckinghaus A. Ghosh S. (2009). The NF-κB family of transcription factors and its regulation. Cold Spring Harb. Perspect. Biol. 1:a000034. doi: 10.1101/cshperspect.a000034 20066092 PMC2773619

[ref103] Oswald M. C. W. Garnham N. Sweeney S. T. Landgraf M. (2018). Regulation of neuronal development and function by ROS. FEBS Lett. 592, 679–691. doi: 10.1002/1873-3468.12972, PMID: 29323696 PMC5888200

[ref104] Ou J. Smith R. C. Tobe R. H. Lin J. Arriaza J. Fahey J. W.. (2024). Efficacy of sulforaphane in treatment of children with autism Spectrum disorder: a randomized double-blind placebo-controlled multi-center trial. J. Autism Dev. Disord. 54, 628–641. doi: 10.1007/s10803-022-05784-9, PMID: 36427174

[ref105] Pagano G. Lyakhovich A. Pallardó F. V. Tiano L. Zatterale A. Trifuoggi M. (2024). Mitochondrial dysfunction in fragile X syndrome and fragile X-associated tremor/ataxia syndrome: prospect use of antioxidants and mitochondrial nutrients. Mol. Biol. Rep. 51:480. doi: 10.1007/s11033-024-09415-7, PMID: 38578387 PMC10997711

[ref106] Paulėkas E. Vanagas T. Lagunavičius S. Pajėdienė E. Petrikonis K. Rastenytė D. (2024). Navigating the neurobiology of Parkinson’s: the impact and potential of α-synuclein. Biomedicines. 12:2121. doi: 10.3390/biomedicines12092121, PMID: 39335634 PMC11429448

[ref107] Pawase P. A. Goswami C. Shams R. Pandey V. K. Tripathi A. Rustagi S.. (2024). A conceptual review on classification, extraction, bioactive potential and role of phytochemicals in human health. Future Foods. 9:100313. doi: 10.1016/j.fufo.2024.100313

[ref108] Prochaska H. J. De Long M. J. Talalay P. (1985). On the mechanisms of induction of cancer-protective enzymes: a unifying proposal. Proc. Natl. Acad. Sci. USA 82, 8232–8236. doi: 10.1073/pnas.82.23.8232, PMID: 3934671 PMC391477

[ref109] Prochaska H. J. Santamaria A. B. Talalay P. (1992). Rapid detection of inducers of enzymes that protect against carcinogens. Proc. Natl. Acad. Sci. 89, 2394–2398. doi: 10.1073/pnas.89.6.2394, PMID: 1549602 PMC48664

[ref110] Pu D. Zhao Y. Chen J. Sun Y. Lv A. Zhu S.. (2018). Protective effects of sulforaphane on cognitive impairments and AD-like lesions in diabetic mice are associated with the upregulation of Nrf2 transcription activity. Neuroscience 381, 35–45. doi: 10.1016/j.neuroscience.2018.04.017, PMID: 29684505

[ref111] Qin J. Ma Z. Chen X. Shu S. (2023). Microglia activation in central nervous system disorders: a review of recent mechanistic investigations and development efforts. Front. Neurol. 14:1103416. doi: 10.3389/fneur.2023.1103416 36959826 PMC10027711

[ref112] Qu Y. Yan M. Zhang Q. (2017). Functional regulation of plant NADPH oxidase and its role in signaling. Plant Signal. Behav. 12:e1356970. doi: 10.1080/15592324.2017.1356970, PMID: 28758832 PMC5616152

[ref113] Rahman M. A. Woo J. H. Song Y. Lee S. H. Hasan M. M. Azad M. A. K.. (2022). Heat shock proteins and antioxidant genes involved in heat combined with drought stress responses in perennial Rye grass. Life. 12:1426. doi: 10.3390/life12091426, PMID: 36143461 PMC9506360

[ref114] Robledinos-Antón N. Fernández-Ginés R. Manda G. Cuadrado A. (2019). Activators and inhibitors of NRF2: a review of their potential for clinical development. Oxidative Med. Cell. Longev. 2019:9372182. doi: 10.1155/2019/9372182 PMC6664516 31396308

[ref115] Rojo A. I. McBean G. Cindric M. Egea J. López M. G. Rada P.. (2014). Redox control of microglial function: molecular mechanisms and functional significance. Antioxid Redox Signal 21, 1766–1801. doi: 10.1089/ars.2013.5745 24597893 PMC4186766

[ref116] Ruppersberg J. P. Kitzing E. v. Schoepfer R. (1994). The mechanism of magnesium block of NMDA receptors. Semin. Neurosci. 6, 87–96. doi: 10.1006/smns.1994.1012

[ref117] Rushmore T. H. Morton M. R. Pickett C. B. (1991). The antioxidant responsive element. Activation by oxidative stress and identification of the DNA consensus sequence required for functional activity. J. Biol. Chem. 266, 11632–11639. doi: 10.1016/S0021-9258(18)99004-6, PMID: 1646813

[ref118] Rushmore T. H. Pickett C. B. (1990). Transcriptional regulation of the rat glutathione S-transferase Ya subunit gene. Characterization of a xenobiotic-responsive element controlling inducible expression by phenolic antioxidants. J. Biol. Chem. 265, 14648–14653. doi: 10.1016/S0021-9258(18)77351-1, PMID: 2387873

[ref119] Sandouka S. Shekh-Ahmad T. (2021). Induction of the Nrf2 pathway by sulforaphane is neuroprotective in a rat temporal lobe epilepsy model. Antioxidants. 10:1702. doi: 10.3390/antiox10111702, PMID: 34829573 PMC8615008

[ref120] Shih A. Y. Johnson D. A. Wong G. Kraft A. D. Jiang L. Erb H.. (2003). Coordinate regulation of glutathione biosynthesis and release by Nrf2-expressing glia potently protects neurons from oxidative stress. J. Neurosci. 23, 3394–3406. doi: 10.1523/JNEUROSCI.23-08-03394.2003, PMID: 12716947 PMC6742304

[ref121] Shirai Y. Fujita Y. Hashimoto K. (2012). Effects of the antioxidant sulforaphane on hyperlocomotion and Prepulse inhibition deficits in mice after phencyclidine administration. Clin Psychopharmacol Neurosci. 10, 94–98. doi: 10.9758/cpn.2012.10.2.94, PMID: 23430731 PMC3569145

[ref122] Shirai Y. Fujita Y. Hashimoto R. Ohi K. Yamamori H. Yasuda Y.. (2015). Dietary intake of sulforaphane-rich broccoli sprout extracts during juvenile and adolescence can prevent phencyclidine-induced cognitive deficits at adulthood. PLoS One 10:e0127244. doi: 10.1371/journal.pone.0127244, PMID: 26107664 PMC4479552

[ref123] Simpson D. S. A. Oliver P. L. (2020). ROS generation in microglia: understanding oxidative stress and inflammation in neurodegenerative disease. Antioxidants. 9:743. doi: 10.3390/antiox9080743, PMID: 32823544 PMC7463655

[ref124] Singh K. Connors S. L. Macklin E. A. Smith K. D. Fahey J. W. Talalay P.. (2014). Sulforaphane treatment of autism spectrum disorder (ASD). Proc. Natl. Acad. Sci. 111, 15550–15555. doi: 10.1073/pnas.1416940111, PMID: 25313065 PMC4217462

[ref125] Sparg S. G. Light M. E. van Staden J. (2004). Biological activities and distribution of plant saponins. J. Ethnopharmacol. 94, 219–243. doi: 10.1016/j.jep.2004.05.016 15325725

[ref126] Subedi L. Lee J. H. Yumnam S. Ji E. Kim S. Y. (2019). Anti-inflammatory effect of sulforaphane on LPS-activated microglia potentially through JNK/AP-1/NF-κB inhibition and Nrf2/HO-1 activation. Cells 8:194. doi: 10.3390/cells8020194, PMID: 30813369 PMC6406309

[ref127] Suzuki T. Muramatsu A. Saito R. Iso T. Shibata T. Kuwata K.. (2019). Molecular mechanism of cellular oxidative stress sensing by Keap1. Cell Rep. 28, 746–758. doi: 10.1016/j.celrep.2019.06.047, PMID: 31315052

[ref128] Suzuki T. Takahashi J. Yamamoto M. (2023). Molecular basis of the KEAP1-NRF2 Signaling pathway. Mol. Cells 46, 133–141. doi: 10.14348/molcells.2023.0028, PMID: 36994473 PMC10070164

[ref129] Swerdlow N. R. Braff D. L. Geyer M. A. (2016). Sensorimotor gating of the startle reflex: what we said 25 years ago, what has happened since then, and what comes next. J. Psychopharmacol. 30, 1072–1081. doi: 10.1177/0269881116661075, PMID: 27539931 PMC6036900

[ref130] Tang R. Cao Q. q. Hu S. w. He L. j. Du P. f. Chen G.. (2022). Sulforaphane activates anti-inflammatory microglia, modulating stress resilience associated with BDNF transcription. Acta Pharmacol. Sin. 43, 829–839. doi: 10.1038/s41401-021-00727-z, PMID: 34272506 PMC8976037

[ref131] Tian Y. Wang W. Xu L. Li H. Wei Y. Wu Q.. (2019). Activation of Nrf2/ARE pathway alleviates the cognitive deficits in PS1V97L-Tg mouse model of Alzheimer’s disease through modulation of oxidative stress. J. Neurosci. Res. 97, 492–505. doi: 10.1002/jnr.24357, PMID: 30461032

[ref132] Ul Haq S. Khan A. Ali M. Khattak A. M. Gai W. X. Zhang H. X.. (2019). Heat shock proteins: dynamic biomolecules to counter plant biotic and abiotic stresses. Int. J. Mol. Sci. 20:5321. doi: 10.3390/ijms20215321 31731530 PMC6862505

[ref133] Vauzour D. Buonfiglio M. Corona G. Chirafisi J. Vafeiadou K. Angeloni C.. (2010). Sulforaphane protects cortical neurons against 5-S-cysteinyl-dopamine-induced toxicity through the activation of ERK1/2, Nrf-2 and the upregulation of detoxification enzymes. Mol. Nutr. Food Res. 54, 532–542. doi: 10.1002/mnfr.200900197, PMID: 20166144

[ref134] Villapol S. (2018). Roles of peroxisome proliferator-activated receptor-gamma on brain and peripheral inflammation. Cell. Mol. Neurobiol. 38, 121–132. doi: 10.1007/s10571-017-0554-5, PMID: 28975471 PMC5776063

[ref135] Wilkinson B. L. Landreth G. E. (2006). The microglial NADPH oxidase complex as a source of oxidative stress in Alzheimer’s disease. J. Neuroinflammation 3:30. doi: 10.1186/1742-2094-3-30, PMID: 17094809 PMC1637099

[ref136] Winship I. R. Dursun S. M. Baker G. B. Balista P. A. Kandratavicius L. Maia-de-Oliveira J. P.. (2019). An overview of animal models related to schizophrenia. Can. J. Psychiatr. 64, 5–17. doi: 10.1177/0706743718773728, PMID: 29742910 PMC6364139

[ref137] Woodburn S. C. Bollinger J. L. Wohleb E. S. (2021). The semantics of microglia activation: neuroinflammation, homeostasis, and stress. J. Neuroinflammation 18:258. doi: 10.1186/s12974-021-02309-6, PMID: 34742308 PMC8571840

[ref138] Wu X. Zhao J. Yu S. Chen Y. Wu J. Zhao Y. (2012). Sulforaphane protects primary cultures of cortical neurons against injury induced by oxygen-glucose deprivation/reoxygenation via antiapoptosis. Neurosci. Bull. 28, 509–516. doi: 10.1007/s12264-012-1273-z, PMID: 23054633 PMC5561925

[ref139] Xie T. Belinsky M. Xu Y. Jaiswal A. K. (1995). ARE-and TRE-mediated regulation of gene expression: response to xenobiotics and antioxidants (∗). J. Biol. Chem. 270, 6894–6900. doi: 10.1074/jbc.270.12.6894, PMID: 7896838

[ref140] Xin H. Cui Y. An Z. Yang Q. Zou X. Yu N. (2019). Attenuated glutamate induced ROS production by antioxidative compounds in neural cell lines. RSC Adv. 9, 34735–34743. doi: 10.1039/C9RA03848E, PMID: 35530670 PMC9074000

[ref141] Xing F. Hu Q. Qin Y. Xu J. Zhang B. Yu X.. (2022). The relationship of redox with hallmarks of cancer: the importance of homeostasis and context. Front. Oncol. 12:862743. doi: 10.3389/fonc.2022.862743, PMID: 35530337 PMC9072740

[ref142] Yagishita Y. Fahey J. W. Dinkova-Kostova A. T. Kensler T. W. (2019). Broccoli or sulforaphane: is it the source or dose that matters? Molecules 24:3593. doi: 10.3390/molecules24193593, PMID: 31590459 PMC6804255

[ref143] Zaynab M. Sharif Y. Abbas S. Afzal M. Z. Qasim M. Khalofah A.. (2021). Saponin toxicity as key player in plant defense against pathogens. Toxicon 193, 21–27. doi: 10.1016/j.toxicon.2021.01.009, PMID: 33508310

[ref144] Zhang D. D. Hannink M. (2003). Distinct cysteine residues in Keap1 are required for Keap1-dependent ubiquitination of Nrf2 and for stabilization of Nrf2 by Chemopreventive agents and oxidative stress. Mol. Cell. Biol. 23, 8137–8151. doi: 10.1128/MCB.23.22.8137-8151.2003, PMID: 14585973 PMC262403

[ref145] Zhang Y. Lu L. Jia J. Jia L. Geula C. Pei J.. (2014). A lifespan observation of a novel mouse model: in vivo evidence supports Aβ oligomer hypothesis. PLoS ONE 9:e85885. doi: 10.1371/journal.pone.0085885 24465766 PMC3897547

[ref146] Zhang Y. Talalay P. Cho C. G. Posner G. H. (1992). A major inducer of anticarcinogenic protective enzymes from broccoli: isolation and elucidation of structure. Proc. Natl. Acad. Sci. USA 89, 2399–2403. doi: 10.1073/pnas.89.6.2399, PMID: 1549603 PMC48665

[ref147] Zhang W. Xiao D. Mao Q. Xia H. (2023). Role of neuroinflammation in neurodegeneration development. Signal Transduct. Target. Ther. 8, 1–32. doi: 10.1038/s41392-023-01486-5 37433768 PMC10336149

[ref148] Zimmerman A. W. Singh K. Connors S. L. Liu H. Panjwani A. A. Lee L. C.. (2021). Randomized controlled trial of sulforaphane and metabolite discovery in children with autism Spectrum disorder. Mol. Autism. 12:38. doi: 10.1186/s13229-021-00447-5, PMID: 34034808 PMC8146218

[ref149] Zuiderwijk-Sick E. A. van der Putten C. Timmerman R. Veth J. Pasini E. M. van Straalen L.. (2021). Exposure of microglia to interleukin-4 represses NF-κB-dependent transcription of toll-like receptor-induced cytokines. Front. Immunol. 12:771453. doi: 10.3389/fimmu.2021.771453 34880868 PMC8645606

