# Supplementary material for: Broccoli for the brain: a review of the neuroprotective mechanisms of sulforaphane
Source: Front Cell Neurosci. 2025 Jul 4;19:1601366. doi: 10.3389/fncel.2025.1601366 (PMC12271217; doi:10.3389/fncel.2025.1601366)
Supplement: Supplementary file 1 [file Table_1.docx]

Table 1. List of articles in PubMed results for “Sulforaphane” – Clinical Trials

| **Title** | **Publication Year** | **DOI** | **Category** |
| --- | --- | --- | --- |
| A Phase 1 Randomized, Placebo-Controlled Study Evaluating the Safety, Tolerability, and Pharmacokinetics of Enteric-Coated Stabilized Sulforaphane (SFX-01) in Male Participants | 2025 | 10.1007/s12325-024-03018-1 | **Bioavailability & Pharmacokinetics (Absorption, Metabolism, Kinetics)** |
| Efficacy and Safety of Sulforaphane Added to Antipsychotics for the Treatment of Negative Symptoms of Schizophrenia: A Randomized Controlled Trial | 2025 | 10.4088/JCP.24m15272 | **Neurodevelopmental & Neuropsychiatric Disorders (Autism, Schizophrenia, Cognitive Function, Depression)** |
| Efficacy of Sulforaphane in Treatment of Children with Autism Spectrum Disorder: A Randomized Double-Blind Placebo-Controlled Multi-center Trial | 2024 | 10.1007/s10803-022-05784-9 | **Neurodevelopmental & Neuropsychiatric Disorders (Autism, Schizophrenia, Cognitive Function, Depression)** |
| Improving insulin resistance by sulforaphane via activating the Bacteroides and Lactobacillus SCFAs-GPR-GLP1 signal axis | 2024 | 10.1039/d4fo01059k | **Metabolic Health (Diabetes, Insulin Resistance, Cholesterol, Obesity)** |
| Sulforaphane Supplementation Did Not Modulate NRF2 and NF-kB mRNA Expressions in Hemodialysis Patients | 2024 | 10.1053/j.jrn.2023.08.008 | **Antioxidant & Anti-inflammatory Effects (Nrf2, Oxidative Stress, Airway Inflammation)** |
| Sulforaphane upregulates the mRNA expression of NRF2 and NQO1 in non-dialysis patients with chronic kidney disease | 2024 | 10.1016/j.freeradbiomed.2024.05.034 | **Antioxidant & Anti-inflammatory Effects (Nrf2, Oxidative Stress, Airway Inflammation)** |
| Reply to Curtis, L. Comment on "Magner et al. Sulforaphane Treatment in Children with Autism: A Prospective Randomized Double-Blind Study. Nutrients 2023, 15, 718" | 2024 | 10.3390/nu16050674 | **Excluded (Not a clinical trial)** |
| Paternal Combined Botanicals Contribute to the Prevention of Estrogen Receptor-Negative Mammary Cancer in Transgenic Mice | 2023 | 10.1016/j.tjnut.2023.05.001 | **Cancer Prevention & Chemoprevention (Breast, Prostate, Pancreatic, Oral, Skin)** |
| Sulforaphane Treatment in Children with Autism: A Prospective Randomized Double-Blind Study | 2023 | 10.3390/nu15030718 | **Neurodevelopmental & Neuropsychiatric Disorders (Autism, Schizophrenia, Cognitive Function, Depression)** |
| Accumulation of Sulforaphane and Alliin in Human Prostate Tissue | 2022 | 10.3390/nu14163263 | **Cancer Prevention & Chemoprevention (Breast, Prostate, Pancreatic, Oral, Skin)** |
| Brain Training and Sulforaphane Intake Interventions Separately Improve Cognitive Performance in Healthy Older Adults, Whereas a Combination of These Interventions Does Not Have More Beneficial Effects: Evidence from a Randomized Controlled Trial | 2021 | 10.3390/nu13020352 | **Neurodevelopmental & Neuropsychiatric Disorders (Autism, Schizophrenia, Cognitive Function, Depression)** |
| Efficacy and safety of sulforaphane for treatment of mild to moderate depression in patients with history of cardiac interventions: A randomized, double-blind, placebo-controlled clinical trial | 2021 | 10.1111/pcn.13276 | **Neurodevelopmental & Neuropsychiatric Disorders (Autism, Schizophrenia, Cognitive Function, Depression)** |
| Oral chronic sulforaphane effects on heavy resistance exercise: Implications for inflammatory and muscle damage parameters in young practitioners | 2021 | 10.1016/j.nut.2021.111266 | **Other Clinical Applications (Exercise Performance, Allergies, Autoimmune, Misc.)** |
| Randomized controlled trial of an adjunctive sulforaphane nutraceutical in schizophrenia | 2021 | 10.1016/j.schres.2021.03.018 | **Neurodevelopmental & Neuropsychiatric Disorders (Autism, Schizophrenia, Cognitive Function, Depression)** |
| Randomized controlled trial of sulforaphane and metabolite discovery in children with Autism Spectrum Disorder | 2021 | 10.1186/s13229-021-00447-5 | **Neurodevelopmental & Neuropsychiatric Disorders (Autism, Schizophrenia, Cognitive Function, Depression)** |
| Sulforaphane Bioavailability and Effects on Blood Pressure in Women with Pregnancy Hypertension | 2021 | 10.1007/s43032-020-00439-5 | **Metabolic Health (Diabetes, Insulin Resistance, Cholesterol, Obesity)** |
| The Effect of Broccoli Sprout Extract on Seasonal Grass Pollen-Induced Allergic Rhinitis | 2021 | 10.3390/nu13041337 | **Other Clinical Applications (Exercise Performance, Allergies, Autoimmune, Misc.)** |
| Broccoli sprout supplementation in patients with advanced pancreatic cancer is difficult despite positive effects-results from the POUDER pilot study | 2020 | 10.1007/s10637-019-00826-z | **Cancer Prevention & Chemoprevention (Breast, Prostate, Pancreatic, Oral, Skin)** |
| Effects of probiotics or broccoli supplementation on Helicobacter pylori eradication with standard clarithromycin-based triple therapy | 2020 | 10.3904/kjim.2019.139 | **Gastrointestinal & Helicobacter Pylori (H. pylori, Gut Microbiome, Probiotics)** |
| Study protocol for SFX-01 after subarachnoid haemorrhage (SAS): a multicentre randomised double-blinded, placebo controlled trial | 2020 | 10.1136/bmjopen-2018-028514 | **Other Clinical Applications (Exercise Performance, Allergies, Autoimmune, Misc.)** |
| Sulforaphane as an adjunctive treatment for irritability in children with autism spectrum disorder: A randomized, double-blind, placebo-controlled clinical trial | 2020 | 10.1111/pcn.13016 | **Neurodevelopmental & Neuropsychiatric Disorders (Autism, Schizophrenia, Cognitive Function, Depression)** |
| Sulforaphane Bioavailability and Chemopreventive Activity in Men Presenting for Biopsy of the Prostate Gland: A Randomized Controlled Trial | 2020 | 10.1080/01635581.2019.1619783 | **Cancer Prevention & Chemoprevention (Breast, Prostate, Pancreatic, Oral, Skin)** |
| Bioavailability of Sulforaphane Following Ingestion of Glucoraphanin-Rich Broccoli Sprout and Seed Extracts with Active Myrosinase: A Pilot Study of the Effects of Proton Pump Inhibitor Administration | 2019 | 10.3390/nu11071489 | **Bioavailability & Pharmacokinetics (Absorption, Metabolism, Kinetics)** |
| Broccoli sprout beverage is safe for thyroid hormonal and autoimmune status: Results of a 12-week randomized trial | 2019 | 10.1016/j.fct.2019.02.004 | **Other Clinical Applications (Exercise Performance, Allergies, Autoimmune, Misc.)** |
| Compartmentalization of anti-oxidant and anti-inflammatory gene expression in current and former smokers with COPD | 2019 | 10.1186/s12931-019-1164-1 | **Antioxidant & Anti-inflammatory Effects (Nrf2, Oxidative Stress, Airway Inflammation)** |
| Dose-dependent detoxication of the airborne pollutant benzene in a randomized trial of broccoli sprout beverage in Qidong, China | 2019 | 10.1093/ajcn/nqz122 | **Detoxification & Environmental Pollutants (Airborne Pollutants, Toxins, Xenobiotic Metabolism)** |
| Effects of long-term consumption of broccoli sprouts on inflammatory markers in overweight subjects | 2019 | 10.1016/j.clnu.2018.03.006 | **Metabolic Health (Diabetes, Insulin Resistance, Cholesterol, Obesity)** |
| Evaluation of 2-Thiothiazolidine-4-Carboxylic Acid, a Common Metabolite of Isothiocyanates, as a Potential Biomarker of Cruciferous Vegetable Intake | 2019 | 10.1002/mnfr.201801029 | **Bioavailability & Pharmacokinetics (Absorption, Metabolism, Kinetics)** |
| Transcriptional changes in prostate of men on active surveillance after a 12-mo glucoraphanin-rich broccoli intervention-results from the Effect of Sulforaphane on prostate CAncer PrEvention (ESCAPE) randomized controlled trial | 2019 | 10.1093/ajcn/nqz012 | **Cancer Prevention & Chemoprevention (Breast, Prostate, Pancreatic, Oral, Skin)** |
| Absorption and metabolism of isothiocyanates formed from broccoli glucosinolates: effects of BMI and daily consumption in a randomised clinical trial | 2018 | 10.1017/S0007114518002921 | **Metabolic Health (Diabetes, Insulin Resistance, Cholesterol, Obesity)** |
| Bioavailability of Glucoraphanin and Sulforaphane from High-Glucoraphanin Broccoli | 2018 | 10.1002/mnfr.201700911 | **Bioavailability & Pharmacokinetics (Absorption, Metabolism, Kinetics)** |
| Evaluation of Biodistribution of Sulforaphane after Administration of Oral Broccoli Sprout Extract in Melanoma Patients with Multiple Atypical Nevi | 2018 | 10.1158/1940-6207.CAPR-17-0268 | **Cancer Prevention & Chemoprevention (Breast, Prostate, Pancreatic, Oral, Skin)** |
| Identification of urinary metabolites that correlate with clinical improvements in children with autism treated with sulforaphane from broccoli | 2018 | 10.1186/s13229-018-0218-4 | **Neurodevelopmental & Neuropsychiatric Disorders (Autism, Schizophrenia, Cognitive Function, Depression)** |
| Supplementation of the Diet by Exogenous Myrosinase via Mustard Seeds to Increase the Bioavailability of Sulforaphane in Healthy Human Subjects after the Consumption of Cooked Broccoli | 2018 | 10.1002/mnfr.201700980 | **Bioavailability & Pharmacokinetics (Absorption, Metabolism, Kinetics)** |
| Isothiocyanates are detected in human synovial fluid following broccoli consumption and can affect the tissues of the knee joint | 2017 | 10.1038/s41598-017-03629-5 | **Other Clinical Applications (Exercise Performance, Allergies, Autoimmune, Misc.)** |
| Randomized, split-body, single-blinded clinical trial of topical broccoli sprout extract: Assessing the feasibility of its use in keratin-based disorders | 2017 | 10.1016/j.jaad.2016.10.009 | **Other Clinical Applications (Exercise Performance, Allergies, Autoimmune, Misc.)** |
| A proof-of-concept clinical study examining the NRF2 activator sulforaphane against neutrophilic airway inflammation | 2016 | 10.1186/s12931-016-0406-8 | **Antioxidant & Anti-inflammatory Effects (Nrf2, Oxidative Stress, Airway Inflammation)** |
| A Randomized Controlled Trial of the Effect of Broccoli Sprouts on Antioxidant Gene Expression and Airway Inflammation in Asthmatics | 2016 | 10.1016/j.jaip.2016.03.012 | **Antioxidant & Anti-inflammatory Effects (Nrf2, Oxidative Stress, Airway Inflammation)** |
| Associations between cruciferous vegetable intake and selected biomarkers among women scheduled for breast biopsies | 2016 | 10.1017/S136898001500244X | **Cancer Prevention & Chemoprevention (Breast, Prostate, Pancreatic, Oral, Skin)** |
| Effect of Broccoli Sprouts and Live Attenuated Influenza Virus on Peripheral Blood Natural Killer Cells: A Randomized, Double-Blind Study | 2016 | 10.1371/journal.pone.0147742 | **Other Clinical Applications (Exercise Performance, Allergies, Autoimmune, Misc.)** |
| Lack of Effect of Oral Sulforaphane Administration on Nrf2 Expression in COPD: A Randomized, Double-Blind, Placebo Controlled Trial | 2016 | 10.1371/journal.pone.0163716 | **Antioxidant & Anti-inflammatory Effects (Nrf2, Oxidative Stress, Airway Inflammation)** |
| Phase 1 Study of a Sulforaphane-Containing Broccoli Sprout Homogenate for Sickle Cell Disease | 2016 | 10.1371/journal.pone.0152895 | **Other Clinical Applications (Exercise Performance, Allergies, Autoimmune, Misc.)** |
| Prevention of Carcinogen-Induced Oral Cancer by Sulforaphane | 2016 | 10.1158/1940-6207.CAPR-15-0290 | **Cancer Prevention & Chemoprevention (Breast, Prostate, Pancreatic, Oral, Skin)** |
| A phase II study of sulforaphane-rich broccoli sprout extracts in men with recurrent prostate cancer | 2015 | 10.1007/s10637-014-0189-z | **Cancer Prevention & Chemoprevention (Breast, Prostate, Pancreatic, Oral, Skin)** |
| Absorption and chemopreventive targets of sulforaphane in humans following consumption of broccoli sprouts or a myrosinase-treated broccoli sprout extract | 2015 | 10.1002/mnfr.201400674 | **Bioavailability & Pharmacokinetics (Absorption, Metabolism, Kinetics)** |
| Diet rich in high glucoraphanin broccoli reduces plasma LDL cholesterol: Evidence from randomised controlled trials | 2015 | 10.1002/mnfr.201400863 | **Metabolic Health (Diabetes, Insulin Resistance, Cholesterol, Obesity)** |
| Effect of Sulforaphane in Men with Biochemical Recurrence after Radical Prostatectomy | 2015 | 10.1158/1940-6207.CAPR-14-0459 | **Cancer Prevention & Chemoprevention (Breast, Prostate, Pancreatic, Oral, Skin)** |
| Sulforaphane Bioavailability and Chemopreventive Activity in Women Scheduled for Breast Biopsy | 2015 | 10.1158/1940-6207.CAPR-15-0119 | **Cancer Prevention & Chemoprevention (Breast, Prostate, Pancreatic, Oral, Skin)** |
| Sulforaphane improves the bronchoprotective response in asthmatics through Nrf2-mediated gene pathways | 2015 | 10.1186/s12931-015-0253-z | **Antioxidant & Anti-inflammatory Effects (Nrf2, Oxidative Stress, Airway Inflammation)** |
| Sulforaphane-rich broccoli sprout extract improves hepatic abnormalities in male subjects | 2015 | 10.3748/wjg.v21.i43.12457 | **Other Clinical Applications (Exercise Performance, Allergies, Autoimmune, Misc.)** |
| The Effects of Broccoli Sprout Extract Containing Sulforaphane on Lipid Peroxidation and Helicobacter pylori Infection in the Gastric Mucosa | 2015 | 10.5009/gnl14040 | **Gastrointestinal & Helicobacter Pylori (H. pylori, Gut Microbiome, Probiotics)** |
| Sulforaphane induces ROS mediated induction of NKG2D ligands in human cancer cell lines and enhances susceptibility to NK cell mediated lysis | 2015 | 10.1016/j.lfs.2015.01.026 | **Excluded (Not a clinical trial)** |
| In vivo formation and bioavailability of isothiocyanates from glucosinolates in broccoli as affected by processing conditions | 2014 | 10.1002/mnfr.201300894 | **Bioavailability & Pharmacokinetics (Absorption, Metabolism, Kinetics)** |
| Off-target effects of sulforaphane include the derepression of long terminal repeats through histone acetylation events | 2014 | 10.1016/j.jnutbio.2014.02.007 | **Bioavailability & Pharmacokinetics (Absorption, Metabolism, Kinetics)** |
| Pilot study evaluating broccoli sprouts in advanced pancreatic cancer (POUDER trial) - study protocol for a randomized controlled trial | 2014 | 10.1186/1745-6215-15-204 | **Cancer Prevention & Chemoprevention (Breast, Prostate, Pancreatic, Oral, Skin)** |
| Rapid and sustainable detoxication of airborne pollutants by broccoli sprout beverage: results of a randomized clinical trial in China | 2014 | 10.1158/1940-6207.CAPR-14-0103 | **Detoxification & Environmental Pollutants (Airborne Pollutants, Toxins, Xenobiotic Metabolism)** |
| Sulforaphane treatment of autism spectrum disorder (ASD) | 2014 | 10.1073/pnas.1416940111 | **Neurodevelopmental & Neuropsychiatric Disorders (Autism, Schizophrenia, Cognitive Function, Depression)** |
| Sulforaphane-rich broccoli sprout extract attenuates nasal allergic response to diesel exhaust particles | 2014 | 10.1039/c3fo60277j | **Other Clinical Applications (Exercise Performance, Allergies, Autoimmune, Misc.)** |
| Sulforaphane is not an effective antagonist of the human pregnane X-receptor in vivo | 2013 | 10.1016/j.taap.2012.10.029 | **Bioavailability & Pharmacokinetics (Absorption, Metabolism, Kinetics)** |
| Effect of broccoli sprouts on insulin resistance in type 2 diabetic patients: a randomized double-blind clinical trial | 2012 | 10.3109/09637486.2012.665043 | **Metabolic Health (Diabetes, Insulin Resistance, Cholesterol, Obesity)** |
| Enhancing sulforaphane absorption and excretion in healthy men through the combined consumption of fresh broccoli sprouts and a glucoraphanin-rich powder | 2012 | 10.1017/S0007114511004429 | **Bioavailability & Pharmacokinetics (Absorption, Metabolism, Kinetics)** |
| Influence of broccoli extract and various essential oils on performance and expression of xenobiotic- and antioxidant enzymes in broiler chickens | 2012 | 10.1017/S0007114511005873 | **Detoxification & Environmental Pollutants (Airborne Pollutants, Toxins, Xenobiotic Metabolism)** |
| Isothiocyanate concentrations and interconversion of sulforaphane to erucin in human subjects after consumption of commercial frozen broccoli compared to fresh broccoli | 2012 | 10.1002/mnfr.201200225 | **Bioavailability & Pharmacokinetics (Absorption, Metabolism, Kinetics)** |
| Modulation of the metabolism of airborne pollutants by glucoraphanin-rich and sulforaphane-rich broccoli sprout beverages in Qidong, China | 2012 | 10.1093/carcin/bgr229 | **Detoxification & Environmental Pollutants (Airborne Pollutants, Toxins, Xenobiotic Metabolism)** |
| Bioavailability of Sulforaphane from two broccoli sprout beverages: results of a short-term, cross-over clinical trial in Qidong, China | 2011 | 10.1158/1940-6207.CAPR-10-0296 | **Bioavailability & Pharmacokinetics (Absorption, Metabolism, Kinetics)** |
| LC-MS/MS quantification of sulforaphane and indole-3-carbinol metabolites in human plasma and urine after dietary intake of selenium-fortified broccoli | 2011 | 10.1021/jf201501x | **Bioavailability & Pharmacokinetics (Absorption, Metabolism, Kinetics)** |
| Determination of new biomarkers to monitor the dietary consumption of isothiocyanates | 2010 | 10.3109/1354750X.2010.517567 | **Bioavailability & Pharmacokinetics (Absorption, Metabolism, Kinetics)** |
| Dietary sulforaphane-rich broccoli sprouts reduce colonization and attenuate gastritis in Helicobacter pylori-infected mice and humans | 2009 | 10.1158/1940-6207.CAPR-08-0192 | **Gastrointestinal & Helicobacter Pylori (H. pylori, Gut Microbiome, Probiotics)** |
| Oral sulforaphane increases Phase II antioxidant enzymes in the human upper airway | 2009 | 10.1016/j.clim.2008.10.007 | **Detoxification & Environmental Pollutants (Airborne Pollutants, Toxins, Xenobiotic Metabolism)** |
| Repeated intake of broccoli does not lead to higher plasma levels of sulforaphane in human volunteers | 2009 | 10.1016/j.canlet.2009.04.004 | **Bioavailability & Pharmacokinetics (Absorption, Metabolism, Kinetics)** |
| Bioavailability and kinetics of sulforaphane in humans after consumption of cooked versus raw broccoli | 2008 | 10.1021/jf801989e | **Bioavailability & Pharmacokinetics (Absorption, Metabolism, Kinetics)** |
| Broccoli consumption interacts with GSTM1 to perturb oncogenic signalling pathways in the prostate | 2008 | 10.1371/journal.pone.0002568 | **Cancer Prevention & Chemoprevention (Breast, Prostate, Pancreatic, Oral, Skin)** |
| Consuming broccoli does not induce genes associated with xenobiotic metabolism and cell cycle control in human gastric mucosa | 2007 | 10.1093/jn/137.7.1718 | **Bioavailability & Pharmacokinetics (Absorption, Metabolism, Kinetics)** |
| Effect of meal composition and cooking duration on the fate of sulforaphane following consumption of broccoli by healthy human subjects | 2007 | 10.1017/S0007114507381403 | **Bioavailability & Pharmacokinetics (Absorption, Metabolism, Kinetics)** |
| Safety, tolerance, and metabolism of broccoli sprout glucosinolates and isothiocyanates: a clinical phase I study | 2006 | 10.1207/s15327914nc5501_7 | **Bioavailability & Pharmacokinetics (Absorption, Metabolism, Kinetics)** |
| Effects of glucosinolate-rich broccoli sprouts on urinary levels of aflatoxin-DNA adducts and phenanthrene tetraols in a randomized clinical trial in He Zuo township, Qidong, People's Republic of China | 2005 | 10.1158/1055-9965.EPI-05-0368 | **Cancer Prevention & Chemoprevention (Breast, Prostate, Pancreatic, Oral, Skin)** |
| Glutathione S-transferase M1 polymorphism and metabolism of sulforaphane from standard and high-glucosinolate broccoli | 2005 | 10.1093/ajcn/82.6.1283 | **Bioavailability & Pharmacokinetics (Absorption, Metabolism, Kinetics)** |
| [Effects of sulforaphane-rich broccoli sprouts on H. pylori-infected gastric mucosa] | 2005 |  | **Excluded (Not in English)** |
| Oral broccoli sprouts for the treatment of Helicobacter pylori infection: a preliminary report | 2004 | 10.1023/b:ddas.0000037792.04787.8a | **Gastrointestinal & Helicobacter Pylori (H. pylori, Gut Microbiome, Probiotics)** |
| Disposition of glucosinolates and sulforaphane in humans after ingestion of steamed and fresh broccoli | 2000 | 10.1207/S15327914NC382_5 | **Bioavailability & Pharmacokinetics (Absorption, Metabolism, Kinetics)** |
